# Bayesian adaptive designs for multi-arm trials: an orthopaedic case study

**DOI:** 10.1186/s13063-019-4021-0

**Published:** 2020-01-14

**Authors:** Elizabeth G. Ryan, Sarah E. Lamb, Esther Williamson, Simon Gates

**Affiliations:** 1 Cancer Research UK Clinical Trials Unit, Institute of Cancer and Genomic Sciences, University of Birmingham, Birmingham, B15 2TT UK; 2 Centre for Rehabilitation Research, Nuffield Department of Orthopaedics, Rheumatology & Musculoskeletal Sciences (NDORMS), Botnar Research Centre, University of Oxford, Oxford, OX3 7LD UK; 3 College of Medicine and Health, University of Exeter, Exeter, EX1 2LU UK

**Keywords:** Bayesian adaptive design, Interim analysis, Multi-arm trial, Response adaptive randomisation, Arm dropping, Monitoring, Orthopaedic, Emergency medicine, Randomised controlled trials, Phase III

## Abstract

**Background:**

Bayesian adaptive designs can be more efficient than traditional methods for multi-arm randomised controlled trials. The aim of this work was to demonstrate how Bayesian adaptive designs can be constructed for multi-arm phase III clinical trials and assess potential benefits that these designs offer.

**Methods:**

We constructed several alternative Bayesian adaptive designs for the Collaborative Ankle Support Trial (CAST), which was a randomised controlled trial that compared four treatments for severe ankle sprain. These designs incorporated response adaptive randomisation (RAR), arm dropping, and early stopping for efficacy or futility. We studied the operating characteristics of the Bayesian designs via simulation. We then virtually re-executed the trial by implementing the Bayesian adaptive designs using patient data sampled from the CAST study to demonstrate the practical applicability of the designs.

**Results:**

We constructed five Bayesian adaptive designs, each of which had high power and recruited fewer patients on average than the original designs target sample size. The virtual executions showed that most of the Bayesian designs would have led to trials that declared superiority of one of the interventions over the control. Bayesian adaptive designs with RAR or arm dropping were more likely to allocate patients to better performing arms at each interim analysis. Similar estimates and conclusions were obtained from the Bayesian adaptive designs as from the original trial.

**Conclusions:**

Using CAST as an example, this case study shows how Bayesian adaptive designs can be constructed for phase III multi-arm trials using clinically relevant decision criteria. These designs demonstrated that they can potentially generate earlier results and allocate more patients to better performing arms. We recommend the wider use of Bayesian adaptive approaches in phase III clinical trials.

**Trial registration:**

CAST study registration ISRCTN, ISRCTN37807450. Retrospectively registered on 25 April 2003.

## Background

The traditional phase III trial design generally involves randomising patients to one of two arms, often with equal probability of allocation and using fixed sample sizes. The sample size is calculated using frequentist methods, which involve assuming a particular treatment effect and type I error rate to achieve a particular level of power. Phase III trials generally require large sample sizes, have long duration, and many are declared “unsuccessful” due to a perceived lack of difference between treatment arms [1]. For decades, statisticians have been developing more efficient methods for designing clinical trials, yet the majority of trials continue to use traditional methods.

Adaptive trial designs have the potential to allow trials to answer their questions more efficiently, particularly for multi-arm trials, by enabling design components to be altered based on analyses of accumulated data. Adaptive designs have been encouraged by regulatory bodies (e.g. [2]) and a Consolidated Standards of Reporting Trials (CONSORT) extension for adaptive designs is being developed [3]. All possible decisions and adaptations must be specified before the trial commences, as well as the decision criteria. Potential adaptations in multi-arm trials include: stopping early for high probability of efficacy or futility; arm dropping; and altering the randomisation probabilities between arms, known as outcome or response adaptive randomisation (RAR).

RAR methods are increasingly being proposed as an alternative to equal randomisation (ER) for comparative trials since they allow the treatment allocation probabilities to be updated at each interim analysis based on the accrued outcome data. For instance, the probability of being assigned to an arm could increase when the accumulated outcome data suggest that the treatment arm is superior, and thus maximises the number of patients receiving the better treatment. Advocates of RAR consider it to be more ethical than ER since it can allow more patients to be treated with superior treatments [4–6] whilst providing information about treatment efficacy. However, the use of RAR in phase III trials is controversial, particularly for two-arm trials where it may be inefficient [7, 8].

Arm dropping may be performed in multi-arm trials to remove an arm that does not appear to be effective (e.g. [9]). There is no globally optimal method for patient allocation in multi-arm trials and the choice of method depends on the aims and setting of the trial, as some allocation methods may be more practical than others. It is also advantageous to have planned interim analyses so that if the treatment effect is large and there is a high probability of claiming superiority, or conversely, if the treatment effect is very small or non-existent, then the trial can be stopped early.

Adaptive designs have often been constructed and applied in phase III trials using frequentist approaches (e.g. [10, 11]). Further advantages to trial design and analysis can be gained by using Bayesian methods. The Bayesian approach allows previous information on the treatment effect or response to be incorporated into the design via the prior distribution. The prior distribution is updated as data are observed in the trial to become a posterior distribution. The posterior distribution provides probabilistic statements about the values of various measures of interest, such as the treatment effect, adverse event rates, or arm with the maximum response. For instance, one could obtain from the posterior distribution the probability that the relative risk is less than 1. The prior and posterior distributions also account for uncertainty in the unknown values of the measures of interest. Bayesian approaches may be used for fixed or adaptive designs. The posterior distribution may be updated at any time to incorporate current information and can be used to drive the decisions at the interim analyses, in what we refer to as a “Bayesian adaptive design”.

Bayesian adaptive designs have often been used in early-phase trials, but there are few published phase III trials that have used a Bayesian adaptive approach from the design phase (e.g. [12–14]). In this work we will explore how Bayesian adaptive designs could be constructed for an emergency medicine (orthopaedic) multi-arm trial and examine the potential benefits that these designs may offer.

## Methods

### Case study

The Collaborative Ankle Support Trial (CAST; [15–17]) was a phase III pragmatic, individually randomised controlled trial (RCT) that compared the effectiveness of three types of mechanical ankle support with tubular bandage (control) for patients with severe ankle sprains. The three interventions were the Aircast® ankle brace, the Bledsoe® boot, and a below-knee cast. Patients above 16 years of age with an acute severe ankle sprain who were unable to bear weight, but had no fracture, were recruited from eight emergency departments in England. The primary outcome was the quality of ankle function at 12 weeks post-randomisation as measured by the foot- and ankle-related quality of life (QoL) subscale of the Foot and Ankle Outcome Score (FAOS) [18]. The FAOS QoL subscale ranges from 0 (extreme symptoms) to 100 (no symptoms). Randomisation occurred 2–3 days after the initial visit to the emergency department at a follow-up clinical visit.

The CAST study was designed using frequentist methods and initially planned to have a fixed-sample design, but the sample size was subsequently altered using adaptive sample size re-estimation. A pragmatic approach to estimating the sample size was used, where the Data Monitoring Committee (DMC) reviewed the assumptions regarding the baseline pooled standard deviation of the primary outcome [15]. No comparison of between-group differences was performed during the trial in the original CAST study and no alpha was spent during the study (until the final analysis).

Originally a target sample size of 643 patients was required to provide more than 90% power to detect an absolute difference of 10 in the FAOS QoL, assuming a two-sided type I error rate of 5%, a small to moderate effect size and 20% loss to follow-up [16, 17]. The sample size calculation was based on a standard sample size calculation for a two-sample *t* test with equal variances [16]. The minimal clinically important difference (MCID) in the FAOS QoL subscale was specified as a change between 8 and 10. The aim of this trial was to identify the best arm for treatment of severe ankle sprains to assist in recovery. A limited number of comparisons between the treatment arms were pre-specified in a hierarchical order to protect against the consequences of multiple testing.

After reviewing the underlying assumptions of the sample size calculation, a revised sample size was calculated by the DMC after 100 participants were recruited and an estimated target of 480–520 participants provided at least 80% power to detect the MCID, assuming a two-sided type I error rate of 5% [17].

The CAST study randomised 584 patients: 144 to tubular bandage, 149 to Bledsoe® boot, 149 to Aircast® brace, and 142 to below-knee cast. At 12 weeks post-randomisation, the FAOS QoL was estimated to be 53.5 (95% confidence interval (CI) 48.4–58.6) for the tubular bandage arm. Clinically important benefits were found at 12 weeks in the FAOS QoL with the below-knee cast compared to the tubular bandage (mean difference 8.7; 95% CI 2.4–15.0) and with the Aircast® brace compared to the tubular bandage (mean difference 8; 95% CI 1.8–14.2). The Bledsoe® boot did not offer a clinically important difference over the tubular bandage (mean difference 6.1; 95% CI 0–12.3). These estimates were adjusted for baseline FAOS QoL (standardised using the median as the centre), as well as age and sex.

### Potential adaptations for Bayesian designs

In our Bayesian adaptive designs we want to quickly identify the best performing intervention arm. A secondary aim is to deliver the best therapy to patients within the trial. Our designs will reward better performing arms and remove poorly performing arms. The Bayesian adaptive designs were constructed as one-sided superiority studies as we were interested in demonstrating improvement over control.

To achieve this, the following types of adaptations will be explored: RAR, arm dropping and early stopping for either efficacy or lack of benefit (futility). Below we describe how these adaptive features have been incorporated into the Bayesian designs, as well as the rules with which these adaptations could be implemented. The rules for implementing these adaptations were determined based on the input of clinicians, criteria used in previous studies (e.g. [5, 19]) and the results of simulations which explored a range of clinically relevant values. Decision thresholds (stopping boundaries, arm dropping thresholds, trial success criteria) were also chosen to optimise the probability of trial success, the average number of patients randomised, and the proportion of patients randomised to the best therapy. Stopping boundaries and final analysis success criteria were also chosen to ensure that practically relevant values were used and that the simulated one-sided type I error rate was <2.5%.

The Bayesian adaptive designs were constructed by a statistician (EGR) who was independent of CAST and who was blind to the data and results of the trial until the operating characteristics of the designs had been simulated. The designs were constructed using the CAST protocol, and discussions were held with CAST investigators (SEL and EW) to derive the design parameters, using as similar values to the original study as possible, and to determine how the adaptive features could be incorporated to ensure the designs were practically feasible.

#### Interim analysis schedules and candidate designs

We investigated a range of interim analysis schedules where adaptations could be performed every 50, 100 or 200 patients due for their primary outcome assessment (12 weeks post-randomisation). We note that, operationally, fewer interim analyses are typically preferred. We found that performing RAR or arm dropping more frequently increased the probability of trial success and decreased the average sample size (results not shown), and so we only present the adaptive designs that performed RAR or arm dropping every 50 patients. Assessment of early stopping for efficacy or futility was performed every 200 patients due for their primary outcome assessment in each adaptive design. This was performed less frequently than RAR/arm dropping to control the type I error and reduce operational complexity, particularly for the monitoring committees who may not need to meet for randomisation probability updates or arm dropping decisions. A fixed Bayesian design was also investigated for comparative purposes. For each adaptive design, the maximum sample size was specified to be the same as the original planned sample size (*N* = 643). The Bayesian designs explored are described in Table 1. We note that an interim analysis at 600 patients due for their primary outcome assessment may not provide much additional benefit, unless recruitment is slow, since the maximum sample size may have been randomised by this time. Wason et al. [20] discuss the importance of considering the recruitment rate and follow-up duration when planning the timing of interim analyses in adaptive designs.

**Table 1:** Bayesian adaptive designs explored for the Collaborative Ankle Support Trial

Design	Interim analysis schedule^a^	Arm allocation	Control allocation	Early stopping
1	None	1:1:1:1	Equal to other arms	None
2	Every 200 patients	1:1:1:1	Equal to other arms	Efficacy or futility every 200 patients
3	Every 50 patients	Arm dropping assessed at each interim analysis	Equal to other arms	Efficacy or futility every 200 patients
4	Every 50 patients	RAR at each analysis	Matched to best intervention arm	Efficacy or futility every 200 patients
5	Every 50 patients	RAR at each analysis	Fixed at 40%	Efficacy or futility every 200 patients
6	Every 50 patients	RAR at each analysis	No designated control; tubular bandage is treated as an intervention arm	Efficacy or futility every 200 patients

*RAR* Response adaptive randomisation

^a^At number of patients due for primary outcome follow up (at 12 weeks post-randomisation)

#### Response adaptive randomisation

ER was used prior to the first interim analysis. We wanted to use RAR so that more allocations could be given to the better dose. A number of methods have been proposed for calculating the trial arm allocation probabilities for RAR (e.g. [4, 5, 19, 21, 22]), depending on the aims of the trial. We use the approach given in Equation 2 of [22]. At each interim analysis the randomisation probabilities for the intervention arms were updated to be proportional to the posterior probability that the arm was the best intervention arm:

1 $$ \Pr \left({\pi}_t=\max \Big\{{\pi}_{boot},{\pi}_{brace},{\pi}_{below- knee\ cast}\right\}{\left| data\right)}^{\gamma },\kern3em $$

where *π*_*t*_ is the probability that intervention arm *t* is the best arm and *π*_*boot*_, *π*_*brace*_, *π*_*below* − *knee cast*_ are the probabilities that each of the intervention arms are the best. This probability was raised to the power *γ* to avoid extreme randomisation probabilities. We chose *γ* = 0.6 based on the operating characteristics it produced. The randomisation probabilities were then adjusted to sum to 1. Enrolment was suspended to arms that had a randomisation probability <0.1 (and the randomisation probabilities were re-adjusted to sum to 1). The suspended arm(s) could re-enter the randomisation allocation at later interim analyses if the randomisation probabilities crossed above the threshold.

Similar to Viele et al. [23], we explored designs that employed different approaches for control arm allocation in RAR. First, we simulated trials in which the control allocation was matched to the intervention arm with the highest probability of allocation. This maximises the power for the comparison of the best arm to the control. We then assumed a fixed control allocation of approximately 40%, which may be preferred for logistical reasons. Various fixed allocations for the control were explored via simulation and the allocation of 40% was chosen based on the resulting power it produced (results not shown). A similar optimal control allocation has been previously found [23, 24]. Finally, we explored a design in which the control arm (tubular bandage) allocation varied according to its probability of being the best arm. In this design, all arms were considered as interventions, and recruitment to the tubular bandage arm could be suspended if it had a low probability of being the best arm (as for the other arms).

#### Arm dropping

We also investigated the use of permanent arm dropping, where an arm could be dropped if it had a low posterior probability (<10%) of being the best arm at an interim analysis. In the arm dropping designs, the control arm could not be dropped, but any intervention arm could be dropped. If an arm was dropped, the randomisation block size was reduced, but the overall maximum sample size was kept the same. Equal allocation was used for the remaining arms.

#### Early stopping for efficacy or futility

Early stopping for efficacy and futility was assessed at interim analyses performed when 200, 400 and 600 patients were due for their primary outcome assessment visit (12 weeks post-randomisation) in all adaptive designs.

For most of the adaptive designs explored (designs 2–5; Table 1), we allowed early stopping for efficacy if there was a fairly large posterior probability of there being an MCID of 8 between the best intervention arm and the tubular bandage in the primary outcome (Eq. 2) and if there was a high probability (>90%) that the arm is the best arm (Eq. 3):

2 $$ \Pr \left({\theta}_{\mathrm{B} est}-{\theta}_{tubular\ bandage}>8| data\right)>{S}_i $$

3 $$ \mathrm{and}\Pr \left({\pi}_t=\max \Big\{{\pi}_{boot},{\pi}_{brace},{\pi}_{below- knee\ cast}\right\}\left| data\right)>0.9 $$

where *θ*_*Best*_ and *θ*_*tubular bandage*_ are the FAOS QoL scores at 12 weeks for the best intervention arm and the tubular bandage, respectively, and *S*_*i*_ is the stopping boundary for efficacy at interim analysis *i* for the comparison of the best arm to the tubular bandage.

Both criteria in Eqs. 2 and 3 must be met for the trial to stop early for efficacy. The *S*_*i*_ values used were 0.75, 0.7 and 0.6 for interim analyses performed at 200, 400 and 600 patients due for their primary outcome visit, respectively. These values were used for designs 2–5 (Table 1). The stopping boundaries were chosen to ensure acceptable power and were clinically relevant values.

We also defined success criteria for the trial at the final analysis to enable the type I error and power to be calculated and compared across the designs. At the final analysis, the trial was declared successful for designs 1–5 if:

4 $$ \Pr \left({\theta}_{Best}-{\theta}_{tubular\ bandage}>8\ | data\right)>0.5 $$

If this criterion was not met, then the trial was declared unsuccessful.

For designs 2–5, early stopping for statistical futility was based on having a small posterior probability that the best arm is better than the tubular bandage:

5 $$ \Pr \left({\theta}_{Best}>{\theta}_{tubular\ bandage}\ |\  data\right)<0.05 $$

Design 6 (Table 1) used RAR where allocation to the tubular bandage arm could vary according to its probability of being the best arm. This design focussed on identifying the best arm overall with a high probability rather than looking for an MCID between intervention arms and the tubular bandage arm. The motivation behind design 6 was to reduce allocation to poorly performing arms, including the tubular bandage arm. Early stopping for efficacy or futility was based on the probability of being the best arm, evaluated at the best arm:

$$ \Pr \left({\pi}_t=\max \Big\{{\pi}_{tubular\ bandage},\kern0.5em {\pi}_{boot},{\pi}_{brace},{\pi}_{below- knee\  ca\mathrm{s}t}\right\}\left| data\right), $$

where *t* is the best arm. If this probability was <0.1 at interim analyses performed at 200, 400 or 600 patients, then the trial was stopped early for futility. If this probability was >0.975 at 200 patients, >0.95 at 400 patients, or >0.925 at 600 patients, then the trial was stopped early for efficacy. The trial was deemed to be successful at the final analysis if this probability was >0.9. These stopping boundaries were chosen to produce high power and (1-sided) type I error <2.5%.

### Simulation settings

Simulations of the designs were performed in the Fixed and Adaptive Clinical Trial Simulator (FACTS; version 6.2) [25] software so that the operating characteristics of each design could be studied. We used a recruitment rate of 5 patients/week and assumed it took 12 weeks to reach this recruitment rate. We also explored recruitment rates of 25 and 56 patients/week (assuming it took 12 weeks to reach these recruitment rates). We used the same dropout rate that the original study design assumed (20%).

The posterior distribution was estimated for each treatment arm, and the FAOS QoL estimates at 12 weeks were adjusted for the baseline scores using a linear model. The (unadjusted) mean response for each arm was assumed to be normally distributed with a mean FAOS QoL of 50 and a standard deviation of 20. The variance of the FAOS QoL was modelled using an inverse-gamma distribution, where the central variance value was assumed to be 20^2^ and a weight of 1 was used (giving α = 0.5, β = 200). There was little previous information available at the time that the CAST study was designed and so we relied upon the opinions of clinicians in forming the prior distributions. Further details on the model and priors used are given in Additional file 1.

Prior to the start of the CAST study there was uncertainty regarding the effect size and FAOS QoL values, and so we simulated a range of different true effect size scenarios for each design. The different scenarios explored for the primary outcome in each arm are given in Table 2.

**Table 2:** Scenarios explored for Bayesian designs

Scenario	Mean control/tubular bandage FAOS QoL (SD)	Mean boot FAOS QoL (SD)	Mean brace FAOS QoL (SD)	Mean below-knee cast FAOS QoL (SD)
Null	50 (20)	50 (20)	50 (20)	50 (20)
One works, 10 more	50 (20)	50 (20)	50 (20)	60 (20)
One works, 5 more	50 (20)	50 (20)	50 (20)	55 (20)
Better, Best	50 (20)	55 (20)	60 (20)	65 (20)
One worse, others work	50 (20)	45 (20)	55 (20)	60 (20)
All work, two similar	50 (20)	55 (20)	60 (20)	60 (20)

*FAOS* Foot and Ankle Outcome Score, *QoL* quality of life, *SD* standard deviation

We simulated 10,000 trials for each scenario in Table 2 for each design. The type I error was estimated using the proportion of simulations that incorrectly declared the trial to be successful when no difference was present in the true primary outcome scores (null scenario above). The power was calculated as the proportion of simulations that correctly declared the trial to be successful, when at least one treatment was superior in the true FAOS QoL score.

We wanted to accurately estimate the response of the arm that was chosen to be the best. Some studies have shown that RAR can lead to a larger estimation bias compared to ER (e.g. [8]). To quantify bias in the estimates of the best arm responses, we use the mean square error (MSE) of estimation where the expectation is taken over the space of successful trials since estimation of the best arm is only important in this scenario.

### Virtual re-execution of designs

**Fig. 1:**
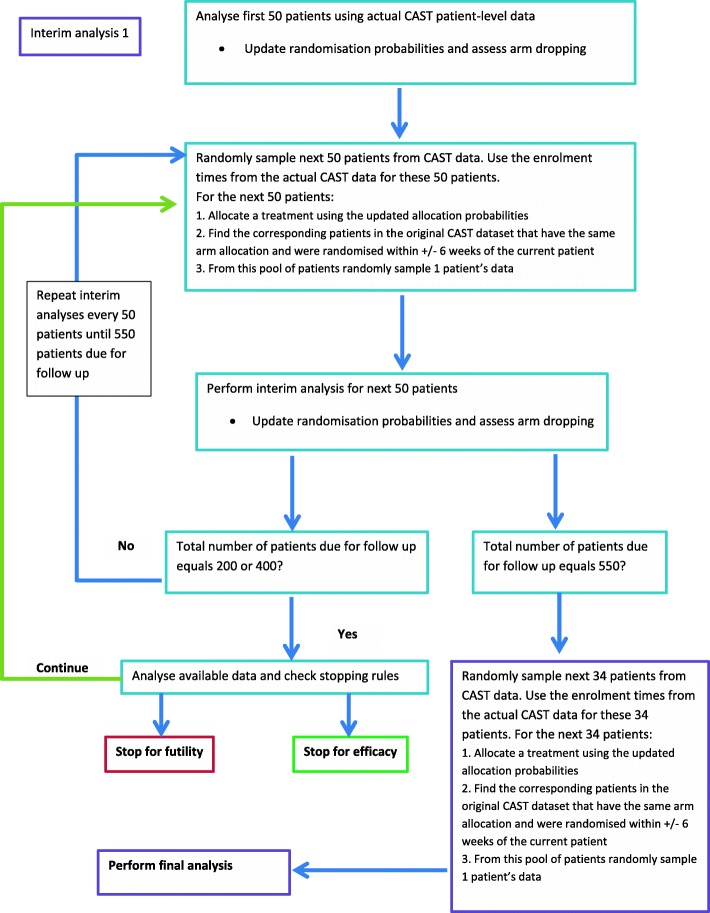
Flow diagram showing the process for the virtual re-execution of designs 3–6. Response adaptive randomisation or arm dropping was performed every 50 patients until the final analysis (at *N* = 584). Early stopping for efficacy or futility was assessed every 200 patients. The process depicted in this figure was repeated 1000 times. CAST Collaborative Ankle Support Trial

A virtual re-execution of the CAST study was performed by implementing the Bayesian designs using the CAST data to illustrate the application and potential benefits of the Bayesian adaptive designs on a real-world trial. We maintained the original enrolment dates for the CAST patients in the re-execution. Since designs 3–6 incorporated arm dropping or RAR every 50 patients, the required allocations for these designs are unlikely to match the allocations that actually occurred in the CAST data. Therefore, at each interim analysis we used the updated randomisation probabilities to obtain allocations for the next 50 patients and then randomly sampled (with replacement) a CAST patient for the re-execution dataset that had a matching treatment allocation and was randomised into the original CAST study within ±6 weeks of the re-execution enrolment date. To avoid bias, for each design the trial was virtually re-executed 1000 times by drawing data from the CAST dataset and performing the interim analyses. A flow diagram of the re-sampling and interim analysis process for designs 3–6 is given in Fig. 1. Further details are given in Additional file 1.

Designs 1 and 2 had fixed arm allocation probabilities throughout the trial, and so we could use the actual CAST data in the virtual executions of these designs without the need for re-sampling. We also used a simplified version of the process described in Fig. 1 to re-sample many datasets from the CAST data to virtually execute designs 1 and 2 so that their results were more comparable to those from designs 3–6. This also enabled us to examine potential gains in efficiency over a range of datasets.

Since the CAST study only recruited 584 patients, we were unable to perform all planned interim analyses. The last interim analysis for early stopping for efficacy/futility occurred at 400 patients. The final analysis occurred once follow-up data had been collected for the 584 patients. The re-executions were performed in R (version 3.5.0; R Foundation for Statistical Computing) and the JAGS package [26] was used to perform the Bayesian analyses. We used a similar approach to Luce et al. [27] to perform the virtual re-executions and re-sampling of patients.

## Results

### Operating characteristics for Bayesian designs

**Fig. 2:**
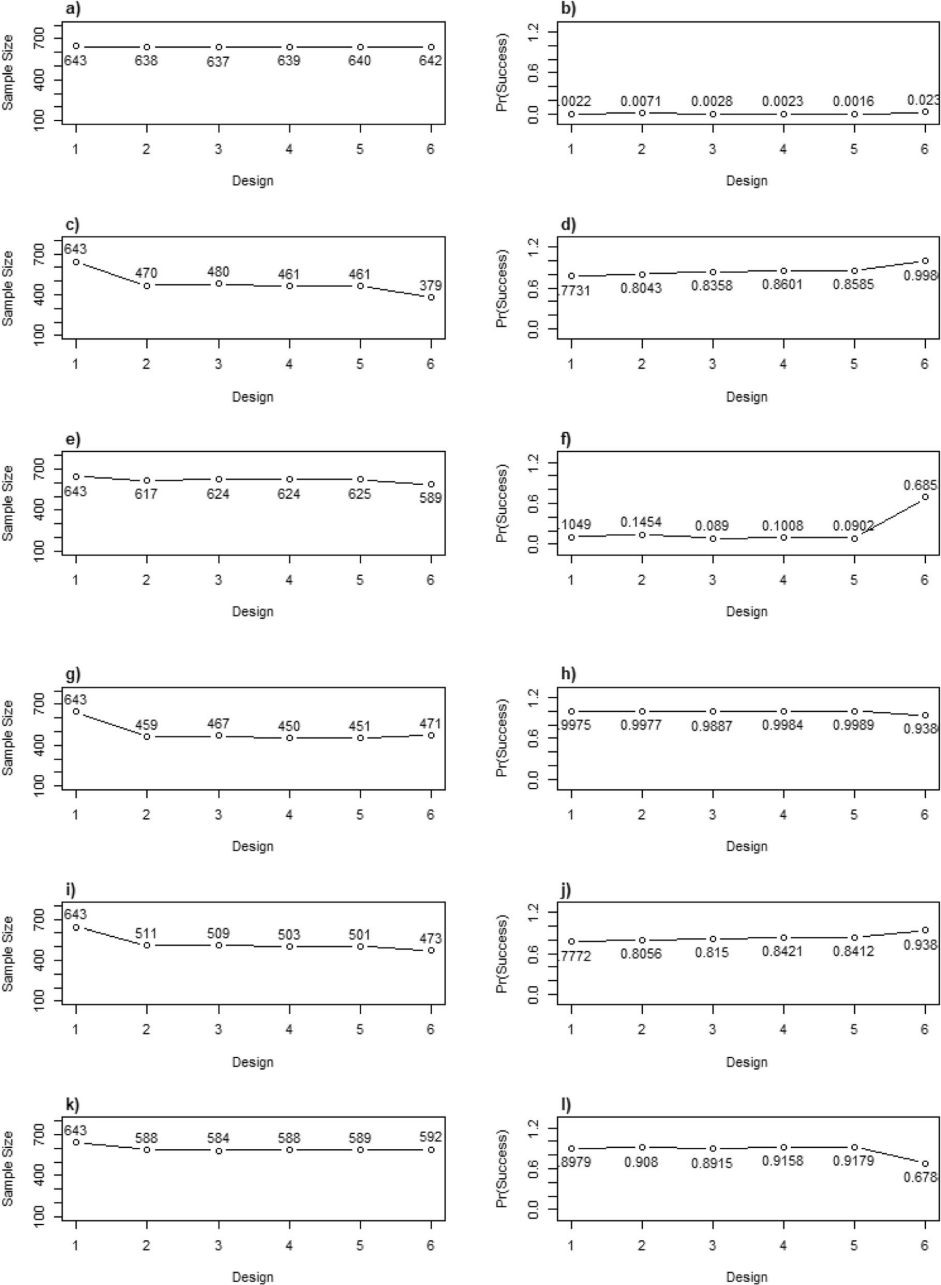
Average sample sizes (**a**, **c**, **e**, **g**, **i**, **k**) and probability of trial success (Pr(Success); **b**, **d**, **f**, **h**, **j**) for each design. Each row represents a different scenario: **a**, **b** “Null” scenario; **c**, **d** “One works, 10 more”; **e**, **f** “One works, 5 more”; **g**, **h** “Better, Best”; **i**, **j** “One worse, others work”; **k**, **l** “All work, two similar”. The type I error is represented in **b**; The power is given in **d**, **f**, **h**, **j**, **l**

**Fig. 3:**
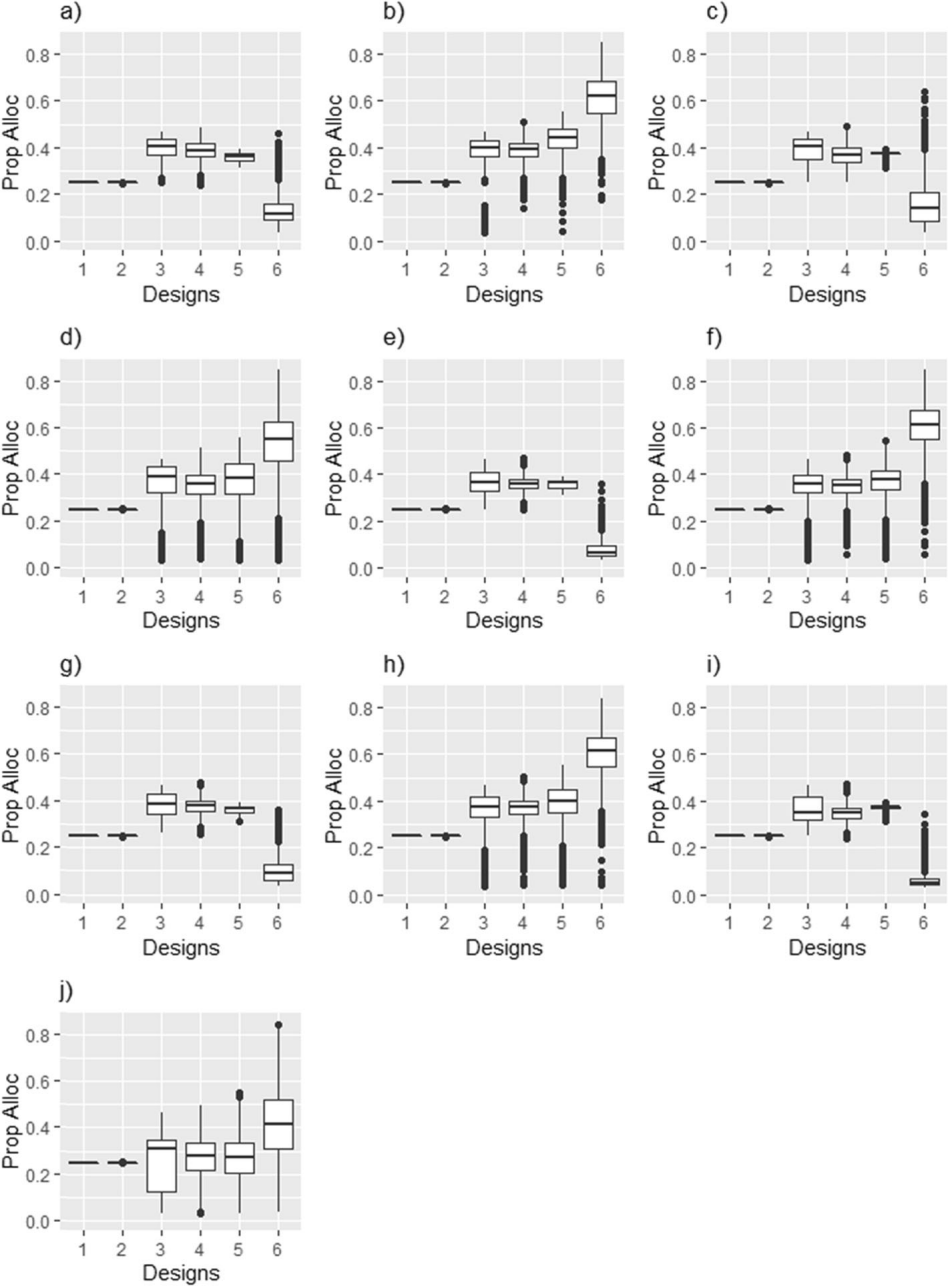
Allocations (Prop Alloc) across 10,000 simulated trials for the tubular bandage arm and true best arm. Each design is represented on the *x* axis. **a** “One works, 10 more” tubular bandage allocation; **b** “One works, 10 more” true best arm allocation; **c** “One works, 5 more” tubular bandage allocation; **d** “One works, 5 more” true best arm allocation; **e** “Better, Best” tubular bandage allocation; **f** “Better, Best” true best arm allocation; **g** “One worse, others work” tubular bandage allocation; **h** “One worse, others work” true best arm allocation; **i** “All work, two similar” tubular bandage allocation; **j** “All work, two similar” true best arm allocation

Select operating characteristics for the Bayesian designs are presented in Table 3 and Fig. 2. Further operating characteristics are given in Additional file 2. Boxplots of the distribution of the allocations to the control/tubular bandage and true best arm for each scenario across the 10,000 simulations are presented in Fig. 3. The effect of using a faster recruitment rate is summarised in Additional file 3.

**Table 3:** Operating characteristics for Bayesian designs for the Collaborative Ankle Support Trial

Scenario	Proportion stopping early for efficacy	Proportion stopping early for futility	MSE	Mean proportion allocated to control	Mean proportion allocated to boot	Mean proportion allocated to brace	Mean proportion allocated to below-knee cast
Null (50, 50, 50, 50)							
Design 1	NA	NA	NA	0.25	0.25	0.25	0.25
Design 2	0.0063	0.013	NA	0.25	0.25	0.25	0.25
Design 3	0.0025	0.0248	NA	0.36	0.21	0.21	0.21
Design 4	0.0022	0.0125	NA	0.33	0.22	0.22	0.22
Design 5	0.0015	0.0134	NA	0.37	0.21	0.21	0.21
Design 6	0.0117	0	NA	0.25	0.25	0.25	0.25
One arm works, 10 more (50, 50, 50, 60)							
Design 1	NA	NA	2.77	0.25	0.25	0.25	0.25
Design 2	0.732	0	5.03	0.25	0.25	0.25	0.25
Design 3	0.6919	0.0022	3.68	0.40	0.11	0.11	0.39
Design 4	0.796	0	3.56	0.39	0.11	0.11	0.39
Design 5	0.7909	0	3.29	0.36	0.10	0.10	0.44
Design 6	0.9972	0	2.34	0.13	0.13	0.13	0.61
One arm works, 5 more (50, 50, 50, 55)							
Design 1	NA	NA	NA	0.25	0.25	0.25	0.25
Design 2	0.1091	0.0015	NA	0.25	0.25	0.25	0.25
Design 3	0.0624	0.0052	NA	0.39	0.13	0.13	0.35
Design 4	0.0733	0.0008	NA	0.37	0.14	0.14	0.35
Design 5	0.0677	0.001	NA	0.37	0.13	0.13	0.37
Design 6	0.5654	0	NA	0.15	0.15	0.15	0.54
Better best (50, 55, 60, 65)							
Design 1	NA	NA	3.29	0.25	0.25	0.25	0.25
Design 2	0.7953	0	5.11	0.25	0.25	0.25	0.25
Design 3	0.6843	0.0001	4.16	0.37	0.10	0.19	0.34
Design 4	0.8177	0	4.05	0.36	0.11	0.19	0.35
Design 5	0.8069	0	3.86	0.36	0.10	0.18	0.37
Design 6	0.8982	0	1.95	0.07	0.10	0.22	0.61
One worse, others work (50, 45, 55, 60)							
Design 1	NA	NA	2.96	0.25	0.25	0.25	0.25
Design 2	0.6341	0	5.10	0.25	0.25	0.25	0.25
Design 3	0.6123	0.0005	3.86	0.38	0.07	0.18	0.36
Design 4	0.6872	0	3.67	0.38	0.07	0.18	0.37
Design 5	0.6856	0	3.43	0.36	0.07	0.17	0.40
Design 6	0.8972	0	1.95	0.10	0.07	0.22	0.61
All work, two similar (50, 55, 60, 60)							
Design 1	NA	NA	3.39	0.25	0.25	0.25	0.25
Design 2	0.2701	0	5.24	0.25	0.25	0.25	0.25
Design 3	0.2692	0.0004	3.87	0.36	0.11	0.27	0.26
Design 4	0.2744	0.2744	3.73	0.35	0.12	0.27	0.27
Design 5	0.2744	0	3.54	0.37	0.10	0.26	0.27
Design 6	0.5493	0	2.93	0.06	0.12	0.41	0.41

*MSE* mean square error, *NA* not applicable

The Bayesian adaptive designs generally offered a decreased average sample size and increased power/probability of trial success across the scenarios explored, compared to the Bayesian fixed design (design 1). The Bayesian adaptive designs only offered small savings in the average sample size for the null scenario (*N*_average_ = 637–642 compared to *N* = 643 in the fixed design) since we used stringent futility stopping rules. For designs 1–5, which used efficacy criteria based on the probability of an MCID, the simulated type I error was approximately 0. Whilst the efficacy stopping boundaries could have been lowered to produce a type I error closer to 2.5%, we felt that lower thresholds for efficacy stopping would not have been practically sensible nor accepted by the clinical community. Designs 2–5 offered modest reductions in the average sample size when a difference of 5 was assumed between the tubular bandage and the best intervention arm, with design 2 producing the lowest average sample size (*N*_average_ = 617) and highest probability of trial success (14.54%).

Designs 4 and 5, which performed RAR, tended to produce the lowest average sample sizes and highest power for the scenarios where one arm was clearly performing best and had an MCID, in other words “One works, 10 more”, “Better, best”, and “One worse, others work” scenarios. Based on the average sample sizes, these designs offered savings of 142–193 patients across the above-mentioned scenarios whilst maintaining >84% probability of having a successful trial. Designs 2 and 3 were only slightly less efficient for these scenarios. For the scenario where two arms offered the same MCID (“All work, two similar”), designs 2–5 offered similar savings to the sample sizes (*N*_average_ = 584–589) and provided similar probability of trial success (range 89.15–91.79%).

Bayesian design 6, which used RAR and allocated all arms according to their probability of being the best arm, had an acceptable type I error of 2.3%. Design 6 offered large sample size savings for the “One works, 10 more”, “Better, Best” and “One worse, others work” scenarios where the average sample sizes ranged from *N*_average_ = 379 to *N*_average_ = 473 across these scenarios. The probability of trial success was ≥94% for design 6 for these three scenarios. This design offered moderate gains in efficiency for the “One works, 5 more” and “All work, two similar” scenarios, with average sample sizes of *N*_average_ = 589 and *N*_average_ = 592, respectively, and probabilities of trial success of 68.53% and 67.88%, respectively.

We also simulated a scenario where all the intervention arms were inferior to the tubular bandage arm (mean FAOS QoL 50, 45, 45, and 45 for tubular bandage, boot, brace, and below-knee cast, respectively; standard deviation = 20 for each arm). In designs 1–5, all of the simulated trials were declared to be unsuccessful at the final analysis for this scenario and 41.72–58.91% of the simulated trials stopped early for futility (designs 2–5). For this scenario, design 6 had similar results to the “One arm works, 5 more” scenario since it did not consider the tubular bandage to be a control arm and considered one arm to be superior by an FAOS of 5.

A faster recruitment rate was found to decrease the efficiency of the adaptive designs (Additional file 3). Due to the lack of successful trials in the null and “one arm works, 5 more” scenarios for the majority of designs, the MSE was not calculated for these scenarios. The adaptive designs tended to have slightly higher MSE than the fixed design, apart from design 6 which had lower MSE. RAR and arm dropping designs had lower MSE compared to the design that just had early stopping for efficacy or futility (design 2).

Across the designs, the correct selection of the best arm was made in 94–100% of the simulated trials, where at least one arm was superior to control by an MCID (see Additional file 2). From Table 3 and Fig. 3, it can be seen that, on average, more allocations were given to the best arm under designs that incorporated RAR or arm dropping when at least one arm was superior. Equal allocation to the treatment arms was achieved in the null scenario for these designs. Design 6 tended to allocate the highest proportion of patients to the best arm. Designs 3–5 tended to have similar allocations. The designs with RAR or arm dropping (designs 3–6) had a fairly large variation in their allocations to the best arm and the control, and were quite often skewed in their distribution. For design 3, the proportion of arm drops was low for the best arm and high for the other arms (Additional file 2).

### Virtual re-execution of designs

Table 4 presents a summary of the virtual re-execution of the CAST study under each Bayesian design across the 1000 trials that re-sampled the CAST study data.

**Table 4:** Summary of re-executions of the Collaborative Ankle Support Trial using each Bayesian design

	Design 1	Design 2	Design 3	Design 4	Design 5	Design 6
Proportion stopping for efficacy at 200 patients	NA	0.216	0.148	0.166	0.147	0.072
Proportion stopping for efficacy at 400 patients	NA	0.043	0.011	0.017	0.011	0.004
Proportion stopping for futility at 200 patients	NA	0	0	0	0	0
Proportion stopping for futility at 400 patients	NA	0	0	0	0	0
Proportion re-executions declared successful at final analysis	0.855	0.894	0.835	0.865	0.877	0.23
Proportion re-executions tubular bandage (control) declared best at final analysis	0	0	0.001	0	0	0
Proportion re-executions boot declared best at final analysis	0.054	0.057	0.085	0.036	0.021	0.007
Proportion re-executions brace declared best at final analysis	0.437	0.402	0.43	0.451	0.481	0.432
Proportion re-executions below-knee cast declared best at final analysis	0.509	0.541	0.484	0.513	0.498	0.561
Median (IQR) of the posterior mean estimates for tubular bandage	54.25 (52.70–55.68)	53.72 (51.90–55.46)	54.40 (52.99–55.74)	53.91 (52.52–55.30)	53.97 (52.64–55.33)	52.49 (51.68–52.96)
Median (IQR) of the posterior estimates of the difference in means between boot and tubular bandage	5.60 (3.65–7.48)	6.00 (4.02–8.25)	5.65 (3.75–7.56)	4.77 (2.42–6.84)	4.85 (2.58–7.05)	6.42 (3.98–8.15)
Median (IQR) of the posterior estimates of the difference in means between brace and tubular bandage	8.60 (6.52–10.63)	8.66 (6.67–10.89)	7.62 (4.81–10.22)	8.48 (5.65–10.71)	8.67 (5.99–10.73)	9.64 (6.01–11.66)
Median (IQR) of the posterior estimates of the difference in means between below-knee cast and tubular bandage	8.70 (6.86–10.91)	9.69 (7.22–13.29)	8.06 (5.44–10.53)	8.79 (6.57–11.39)–	8.68 (6.58–11.27)	10.57 (8.69–11.78)

*IQR* interquartile range, *NA* not applicable

The results of the re-executions show that the Bayesian adaptive designs recommended early stopping for efficacy in 7.6–25.9% of trial re-executions, with the most frequent early stopping occurring in design 2 which had fixed allocations and only allowed for early stopping of the trial. None of the trial re-executions recommended early stopping for futility since all of the interventions performed better than the tubular bandage. At the final analysis for designs 1–5, 83.5–89.4% of the trials were declared successful. Design 6, where decisions were based on having a high probability of being the best arm, had a low proportion (23%) of trials that were declared successful at the final analysis. This is due to the fact that the brace and below-knee cast had similar primary outcome scores, and both performed well compared to the other arms. Thus, one arm was not often declared superior with a high probability. For each of the Bayesian designs, the below-knee cast was most frequently declared the best arm at the final analysis in the re-executions and thus had the same conclusion as the original trial.

The medians of the posterior estimates for the treatment effects over the 1000 re-executions were generally similar to the original frequentist analysis estimates. Designs 4 and 5 (RAR with control allocation matched to best arm and RAR with fixed control allocation, respectively) had slightly lower estimates of the mean difference between Bledsoe boot and tubular bandage. Design 6 had slightly higher estimates of the mean difference between the ankle brace and tubular bandage, and also between the below-knee cast and tubular bandage. One should also bear in mind that the re-executions were performed on re-sampled data from the original dataset, and so the estimates are likely to vary slightly.

Further summaries of the results and randomisation allocations at each interim analysis for each adaptive design are given in Additional file 4, as well as the results for the re-executions of designs 1 and 2 where no re-sampling of the data was performed. These results show that the randomisation probabilities differed between Bayesian designs 4–6 at each interim analysis, and that these RAR designs often had quite different allocations to the CAST study, depending on which arm was “the best” at that interim analysis.

## Discussion

### Summary

In this study we have demonstrated how Bayesian adaptive designs can be constructed for phase III multi-arm RCTs. Using an orthopaedic trial as a case study, we outline the process involved in constructing the designs, describe the adaptive schemes and stopping rules employed, and demonstrate the behaviour of the designs through their operating characteristics across a range of scenarios. We also performed virtual executions of the Bayesian designs using data from the CAST study to demonstrate the decisions that would be made using the Bayesian designs and the trial data. Through use of the Bayesian adaptive approach we were able to make decisions about whether to stop the trial early based on the probability of having an MCID, update the randomisation allocations according to the probability of being the best arm, and suspend recruitment to arms that had a low probability of being the best.

Based on the operating characteristics, the use of Bayesian adaptive designs for this case study generally increased the power and decreased the average sample size compared to a fixed design. The use of RAR generally offered slightly increased power and slightly smaller average sample sizes compared to adaptive designs that employed equal randomisation allocations at each interim analysis (with or without arm dropping) when it was assumed that one arm offered an MCID. Small sample size savings were obtained when no effect or a small effect was assumed to occur, and when two arms were assumed to have an MCID. All designs had low type I error and high probabilities to detect an MCID in at least one arm when it was assumed that one arm was superior and had an MCID. The correct selection of the best arm was made in 94–100% of the simulated trials where at least one arm was superior to control with an MCID. Use of RAR or arm dropping produced simulated trials that gave more allocations to the best arm when at least one arm was superior. Equal allocation occurred when the arms had approximately the same primary outcome scores.

Design 6, the decisions of which were made based on the probability of being the best arm, showed that it could potentially produce large savings in sample size for scenarios where one arm was clearly superior and had an MCID, whilst maintaining high power. However, this design was less efficient when two arms showed a similar improvement compared to the other arms since it was unable to declare a single arm as superior with a high probability. Design 6 had different objectives and decision criteria to the other Bayesian designs, and so care should be taken when choosing a preferred design since the designs are tailored to the aims of the investigators. Criteria such as those used in Design 6 are useful for multi-arm studies in which the investigators want to order the treatments by effectiveness.

The virtual executions of the Bayesian designs using the CAST data showed that early stopping for efficacy only occurred in a small proportion of trials and that no trials stopped early for futility. At the final analysis, >80% of the trials were declared successful in the 1000 executions of designs 1–5. When design 6 was executed 1000 times using the resampled trial data, only 23% of the trials were declared successful at the final analysis since both the brace and below-knee cast performed similarly well and a “best arm” was not declared with a high probability. A benefit of design 6 was that the tubular bandage arm, which was the control arm in the other designs, had smaller allocation probabilities which allowed more allocations to better performing arms. The below-knee cast was most often declared the best arm at the final analysis in the re-executions, and so the Bayesian designs led to the same conclusion as the original trial. If we had known a priori that two arms were likely to perform similarly well, then we would have chosen different success criteria. These results also reflect the problem of dichotomy at a final analysis—if we just reported posterior probabilities of a treatment benefit or MCID then the trial would likely have been viewed more optimistically.

The decisions made at the interim and final analyses of the Bayesian designs were driven by the primary outcome. We did not incorporate other outcomes and are not intending that the conclusions generated in this re-execution be used to inform clinical practice or to alter the conclusions of the original study.

Recruitment can often be challenging in clinical trials, causing delays in their delivery. Approaches which reduce the sample size whilst maintaining high power to determine the effect of interventions should be welcomed by study teams to assist them in completing recruitment on time and within budget.

### Limitations

Adaptive designs have great promise for producing trials with better operating characteristics but present a number of practical challenges. Korn and Freidlin [28] provide a summary of some of the advantages and disadvantages of different adaptive design elements. Wason et al. [20] provide a discussion around the situations in which adaptive designs are and are not useful, and some of the logistical challenges they present.

Adaptive designs require a larger amount of expertise and work to build and evaluate potential designs compared to fixed designs, often involving extensive simulations, and may take more effort to obtain approval from review boards. However, the use of the simulations forces the study team to consider the effects of faster/slower recruitment, follow-up length, smaller effect sizes than anticipated, or higher/lower response rates than anticipated on the operating characteristics of the adaptive designs. Thus, the simulations required by adaptive designs allow study teams to anticipate the effects of differing trial conditions, which are often not considered when using traditional designs.

Adaptive designs can also be more complicated to implement. Performance of the interim analyses and making the required adaptations is dependent on being able to collect, enter, clean and analyse data in a timely manner, and alter the randomisation system with ease. This requires the trial management team, statisticians, programming teams and trial treatment providers/intervention suppliers to be responsive to changes that need to be made. Otherwise, the adaptive designs may lose their gains in efficiency. Timely data entry may be difficult for orthopaedic studies where primary outcomes may be obtained from patient-completed questionnaires that are collected within a 2- to 4-week window of a long follow-up period. The rapid changes required may not be possible in all trial settings.

The interim analyses also need to be adequately spaced to allow time for DMCs and Trial Steering Committees (TSCs) to meet. Statistically, more frequent interim analyses generally produce better operating characteristics for designs that use RAR or arm dropping (e.g. [29]), but frequent interim analyses may not always be practical. The DMC/TSC may not necessarily need to meet for every interim analysis, for example for RAR adaptations, but would need to meet for stopping decisions.

The types of adaptations that can be made to multi-arm trials are situation-dependent. RAR presents difficulties in being able to anticipate and arrange for the delivery of treatments. The original CAST study design, which had fixed allocations, allowed the supply of treatment arms (including the supply of staffing) to be planned more easily than a design with RAR. RAR may not always be possible due to restrictions on resources for delivering the treatments or delays in collecting the primary outcome data. Closure of arms may be practically easier to achieve, particularly for a trial such as CAST for which there need to be sufficient supplies of each treatment available as well as staff proficient in their administration. Whilst early stopping of trials may have benefits for funding agencies, academic trial investigators often do not wish to terminate trials early due to potential loss of research income and staff retention. Changes in funding models are likely to be required to fully take advantage of innovation in trial design, such as a minimum study time funded with a mechanism to release funding if full study time is required. Additionally, trials that stop early may have little information on the long-term effects of treatment, on secondary outcomes, or on cost-effectiveness. They are also likely to produce less precise estimates of the treatment effects. Gallo [30] provides further discussion on some of the operational challenges in adaptive design implementation.

Multi-arm, multi-stage (MAMS) designs are another method for improving the efficiency and ethics in multi-arm trials (with a common control) where experimental arms may be dropped at pre-planned analysis points if they show insufficient evidence of effectiveness. Wason and Trippa [6] showed that Bayesian designs with RAR are more efficient than MAMS designs when there is a superior experimental arm, but that MAMS designs perform slightly better if none of the experimental arms are effective. They also showed that the operating characteristics for the RAR designs were less sensitive than MAMS designs to changes in the amount of primary outcome data available at the interim analyses to the original planned number.

The use of RAR remains controversial and some of its properties are not well understood by clinicians. RAR has its greatest potential in multi-arm trials but has limited usefulness in two-armed trials [7, 31]. Adaptive designs are more susceptible to changes in patient population over time. Designs with RAR have been shown to be robust to moderate changes in patient population, and certain RAR rules have been shown to be effectively unaffected by time trends [32, 33], but adaptive designs are not appropriate if the patient population changes dramatically during the trial. When evaluating adaptive designs, simulation is required to illustrate the operating characteristics and potential benefits, and investigate potential biases introduced by each adaptive feature.

Fairly short follow-up times, relative to the planned recruitment duration, are required for adaptive designs to offer improved efficiency. Adaptive designs are difficult to implement for very fast recruitment rates, particularly for studies that have relatively longer follow-up periods since less information will be available at each interim analysis [6, 20]. We also found that a faster recruitment rate decreased the efficiency of the adaptive designs. This poses difficulties for phase III trials, such as those performed in orthopaedics/rehabilitation, since the primary outcome is often based on long-term measures, and it may be difficult to design adaptive trials without extending the time frame of recruitment to allow for the interim analyses and potential adaptations to occur. Thus, there may be a trade-off in reduced sample size but increased recruitment time (at a slower recruitment rate) for some adaptive trial design contexts.

In this work we virtually executed each of the proposed Bayesian designs using trial data to illustrate their practical applicability. However, in reality, one design would have been chosen and implemented, depending on its operating characteristics, practical restraints and the aims of the trial. Although we tried to ensure that the statistician (EGR) remained blind to the trial results until the design operating characteristics had been obtained via simulations, the study clinicians were involved in discussions around the prior distributions and stopping criteria. It is difficult to completely remove hindsight bias in these historical case studies.

When virtually executing the designs that incorporated arm dropping or RAR, re-sampling from the original trial data was required to obtain the required randomisation allocations. This may lead to an underestimation of the uncertainty in the results [5]. We addressed this by re-executing the CAST study 1000 times and re-sampled patients within each trial. If different datasets had been used, different conclusions may have been obtained using these designs.

We did not simulate the decision making process of a DMC/TSC. We have assumed that the decision-making process was driven by the primary outcome, but the DMC/TSC would also examine safety data and any relevant external evidence. Whilst the role of these committees is to ensure that the study protocol is accurately followed, they may also need to make deviations to ensure patient safety. For example, RAR may recommend increasing the allocation probability to an arm that has a higher rate of adverse events—an event that was not accounted for in the RAR algorithm. Alterations to the previously defined adaptations can lead to unknown operating characteristics.

The Bayesian adaptive designs were constructed as one-sided superiority studies, whereas the original CAST study was a two-sided trial. We were interested in demonstrating improvement over a much cheaper control and felt that a DMC would be unlikely to continue enrolment into a poorly performing comparator just to show it is worse. Under most of our Bayesian adaptive designs, if an intervention arm performed poorly it would be dropped or have a very low probability of allocation. Harm may or may not be reflected in the FAOS QoL score, but the DMC could intervene if any arms were causing harm.

The designs presented here are situation-specific and have been tailored to the clinical situation and aims of the CAST study. The definition of a successful trial and the level of sufficient evidence required to make decisions will differ between researchers and stakeholders, and will depend on the consequences of the actions that may be taken. The designs and findings from this work will not generalise to all phase III RCTs, but similar approaches can be used to construct Bayesian adaptive designs. We recommend that simulations are used to study the impact of each type of adaptive component on the operating characteristics when constructing Bayesian adaptive designs for multi-arm trials.

One of the potential barriers to using Bayesian adaptive designs in practice is the computational time and resources that are required to construct the designs. Trialists or statisticians less familiar with Bayesian methods may not have the time or knowledge to program their own Bayesian adaptive designs, and commercial solutions such as FACTs may not be available to all. A review of available software and code for adaptive clinical trial designs is provided by Grayling and Wheeler [34].

## Conclusions

To enable phase III trials to achieve their aims, more efficient methods are required. Innovation in clinical trial design is extremely important as it can potentially improve the efficiency, quality of knowledge gained, cost and safety of clinical trials. In this work we have demonstrated how Bayesian adaptive trials can be designed and implemented for multi-arm phase III trials. Using a published example from orthopaedic medicine, we highlight some of the benefits of these designs, particularly for multi-arm trials.

## Supplementary information

**Additional file 1.** Additional information on models, priors and the re-execution process.

**Additional file 2.** Additional operating characteristics.

**Additional file 3.** Faster recruitment rates.

**Additional file 4.** Virtual execution of Bayesian designs.

## Data Availability

The data used in this study were generated as part of the CAST study. Requests to share individual, de-identified participant data, aggregated data, data dictionaries, and other study documents from this study should be sent to the CAST Chief Investigator (SEL). Data sharing requests will be assessed on their individual merits. The FACTS files used to simulate the Bayesian adaptive designs are publicly available at https://github.com/egryan90/Bayesian-adaptive-designs-for-CAST-study-Ryan-et-al.-2019.

## Abbreviations

CAST: Collaborative Ankle Support Trial
CI: Confidence interval
DMC: Data Monitoring Committee
ER: Equal randomisation
FACTS: Fixed and Adaptive Clinical Trial Simulator
FAOS: Foot and Ankle Outcome Score
MAMS: Multi-arm, multi-stage
MCID: Minimal clinically important difference
MSE: Mean square error
QoL: Quality of life
RAR: Response adaptive randomisation
RCT: Randomised controlled trial
TSC: Trial Steering Committee

## References

[1] Arrowsmith J (2011). Trial watch: phase III and submission failures: 2007–2010. Nat Rev Drug Discov.

[2] U.S. Food and Drug Administration. Adaptive designs for clinical trials of drugs and biologics: Guidance for Industry. Rockville: Food and Drug Administration; 2019. Available at: https://www.fda.gov/regulatory-information/search-fda-guidance-documents/adaptive-design-clinical-trials-drugs-and-biologics. Accessed 7 Jan 2020.

[3] Dimairo M, Coates E, Pallmann P, Todd S, Julious SA, Jaki T (2018). Development process of a consensus-driven CONSORT extension for randomised trials using an adaptive design. BMC Med.

[4] Trippa L, Lee EQ, Wen PY, Batchelor TT, Cloughesy T, Parmigiani G (2012). Bayesian adaptive randomized trial design for patients with recurrent glioblastoma. J Clin Oncol.

[5] Connor JT, Luce BR, Broglio KR, Ishak KJ, Mullins CD, Vanness DJ (2013). Do Bayesian adaptive trials offer advantages for comparative effectiveness research? Protocol for the RE-ADAPT study. Clin Trials.

[6] Wason JMS, Trippa L (2014). A comparison of Bayesian adaptive randomization and multi-stage designs for multi-arm clinical trials. Stat Med.

[7] Korn EL, Freidlin B (2011). Outcome-adaptive randomization: is it useful?. J Clin Oncol.

[8] Thall P, Fox P, Wathen J (2015). Statistical controversies in clinical research: scientific and ethical problems with adaptive randomization in comparative clinical trials. Ann Oncol.

[9] Wason JMS, Magirr D, Law M, Jaki T (2016). Some recommendations for multi-arm multi-stage trials. Stat Meth Med Res.

[10] Perkins GD, Ji C, Deakin CD, Quinn T, Nolan JP, Scomparin C (2018). A randomized trial of epinephrine in out-of-hospital cardiac arrest. N Engl J Med.

[11] Combes A, Hajage D, Capellier G, Demoule A, Lavoue S, Guervilly C (2018). Extracorporeal membrane oxygenation for severe acute respiratory distress syndrome. N Engl J Med.

[12] Shah PL, Slebos D-J, Cardoso PFG, Cetti E, Voelker K, Levine B (2011). Bronchoscopic lung-volume reduction with Exhale airway stents for emphysema (EASE trial): randomized, sham-controlled, multicentre trial. Lancet.

[13] Reardon MJ, Van Mieghem NM, Popma JJ, Kleiman NS, Søndergaard L, Mumtaz M (2017). Aortic valve replacement in intermediate-risk patients. N Engl J Med.

[14] Nogueira RG, Jadhav AP, Haussen DC, Bonafe A, Budzik RF, Bhuva P (2018). Thrombectomy 6 to 24 hours after stroke with a mismatch between deficit and infarct. N Engl J Med.

[15] Lamb SE, Nakash RA, Withers EJ, Clark M, Marsh JL, Wilson S (2005). Clinical and cost effectiveness of mechanical support for severe ankle sprains: design of a randomised controlled trial in the emergency department. BMC Musculoskelet Disord.

[16] Cooke MW, Marsh JL, Clark M, Nakash R, Jarvis RM, Hutton JL, et al. Treatment of severe ankle sprain: a pragmatic randomised controlled trial comparing the clinical effectiveness and cost-effectiveness of three types of mechanical ankle support with tubular bandage. The CAST trial. Health Technol Assess. 2009;13(13). 10.3310/hta13130. 10.3310/hta13130 19232157

[17] Lamb SE, Marsh JL, Hutton JL, Nakash R, Cooke MW, Collaborative Ankle Support Trial (CAST Group) (2009). Mechanical supports for acute, severe ankle sprain: a pragmatic, multicentre, randomised controlled trial. Lancet.

[18] Roos E, Brandsson S, Karlsson J (2001). Validation of the foot and ankle outcome score for ankle ligament reconstruction. Foot Ankle Int.

[19] Connor JT, Elm JJ, Broglio KR, ESETT and ADAPT-IT Investigators (2013). Bayesian adaptive trials offer advantages in comparative effectiveness trials: an example in status epilepticus. J Clin Epidemiol.

[20] Wason JMS, Brocklehurst P, Yap C (2019). When to keep it simple — adaptive designs are not always useful. BMC Med.

[21] Thall PF, Wathen JK (2007). Practical Bayesian adaptive randomisation in clinical trials. Eur J Cancer.

[22] Wathen JK, Thall PF (2017). A simulation study of outcome adaptive randomization in multi-arm clinical trials. Clin Trials.

[23] Viele K, Broglio K, McGlothlin A, Saville BR. Comparison of methods for control allocation in multiple arm studies using response adaptive randomisation. Clin Trials. 2019. 10.1177/1740774519877836 (epub ahead of print). 10.1177/1740774519877836 31630567

[24] Dunnett C (1955). A multiple comparison procedure for comparing several treatments with a control. J Amer Stat Assoc.

[25] Fixed and Adaptive Clinical Trial Simulator (FACTS). Version 6.2. Berry Consultants: Austin; 2018. https://www.berryconsultants.com/software/.

[26] Plummer M (2003). JAGS: a program for analysis of Bayesian graphical models using Gibbs sampling. Proceedings of the 3rd International Workshop on Distributed Statistical Computing (DSC 2003).

[27] Luce BR, Connor JT, Broglio KR, Mullins CD, Ishak KJ, Saunders E (2016). Using Bayesian adaptive trial designs for comparative effectiveness research: a virtual trial execution. Ann Intern Med.

[28] Korn EL, Freidlin B (2017). Adaptive clinical trials: advantages and disadvantages of various adaptive design elements. J Natl Cancer Inst.

[29] Jiang Y, Zhao W, Durkalski-Mauldin V (2017). Impact of adaptation algorithm, timing, and stopping boundaries on the performance of Bayesian response adaptive randomization in confirmative trials with a binary endpoint. Contemp Clin Trials.

[30] Gallo P (2006). Operational challenges in adaptive design implementation. Pharm Stat.

[31] Berry DA (2011). Adaptive clinical trials: the promise and the caution. J Clin Oncol.

[32] Cook JD. The effect of population drift on adaptively randomized trials: UT MD Anderson Cancer Centre Department of Biostatistics Working Paper Series. Berkeley; 2007. Working Paper 39. https://www.johndcook.com/population_drift.pdf.

[33] Villar SS, Bowden J, Wason J (2018). Response-adaptive designs for binary responses: how to offer patient benefit while being robust to time trends?. Pharm Stat.

[34] Grayling MJ, Wheeler GM (2019). A review of available software for adaptive clinical trial design.

