# Supplementary material for: Bayesian adaptive designs for multi-arm trials: an orthopaedic case study
Source: Trials. 2020 Jan 14;21:83. doi: 10.1186/s13063-019-4021-0 (PMC6961269; doi:10.1186/s13063-019-4021-0)
Supplement: Supplementary file 1 — Additional file 1. Additional information on models, priors and the re-execution process. [file 13063_2019_4021_MOESM1_ESM.docx]

Additional File 1 - Additional information on models, priors and re-execution process

# A1.1 Model and priors

The primary outcome, Foot and Ankle Outcome Score Quality of Life subscale (FAOS QoL) at 12 weeks, was modelled using a baseline-adjusted model. We assumed that the FAOS QoL scores were normally distributed with unknown mean and variance. Thus, the FAOS QoL responses at 12 weeks, denoted by *Y*, were modelled as:

$$Y \sim N(\theta_{t}+ \beta Z, \sigma^{2})$$

The (unadjusted) mean response for arm *t*, $\theta_{t}$, was assumed to be normally distributed: $\theta_{t} \sim N\left( \mu_{t}, v_{t}^{2} \right).$ We assumed that $\mu_{t}=50$ and $v_{t}=20$, based on the opinions of the CAST clinicians. β is a parameter that represents the strength of the relationship between the baseline and 12 week FAOS QoL scores; *Z* is the standardised baseline FAOS QoL score.

An inverse-gamma distribution was assumed for the variance of the response: $\sigma^{2} \sim IG(\frac{\sigma_{w}}{2},\frac{\sigma_{\mu}^{2}\sigma_{w}}{2})$. We assumed the central value of the standard deviation to be $\sigma_{\mu}=20$ with weight $\sigma_{w}=1$, based on the FACTS program documentation (Berry Consultants, 2018). This places a large amount of uncertainty on the variance of the response. A vaguely informative prior was used for the β parameter: $\beta\sim N(14,4^{2})$. This is different to a flat prior, such as $\beta\sim N(0,{100}^{2})$, and was chosen to reflect the degree of influence and variability due to baseline on the final score (Berry Consultants, 2018).

The same priors were used for each treatment arm and the posterior distributions were estimated for each arm. There was little previous information available at the time that the CAST study was designed and so we relied upon the opinions of clinicians in forming the prior distributions. We did not want to use uninformative prior distributions since the FAOS QoL values were restricted to occur between 0 and 100, and therefore used “somewhat informative” priors that were centred around plausible values. Checks of the posterior distributions confirmed that the estimates of the FAOS QoL remained within the range of 0 to 100.

# A1.2 Virtual Re-execution of Designs

A virtual re-execution of the CAST study was performed by implementing the Bayesian designs using the CAST data to illustrate the application and potential benefits of the Bayesian adaptive designs on a real-world trial. We will also investigate if the inferences differ between the results from the Bayesian designs and the original CAST study.

For Designs 3-6, the first 50 patients were analysed in patient order using the actual CAST data. After the first 50 patients were due for primary follow up, RAR or arm dropping could occur. The posterior probability of being the best arm was calculated for each intervention arm at each interim analysis for Designs 3-5, and for all arms for Design 6. From these values, the allocation probabilities for each arm were updated. The relevant posterior probabilities were compared to the arm dropping/suspension criteria, and adjustments were made to the randomisation probabilities if an arm was dropped/suspended (Designs 3-6). This involved ensuring that equal allocation was used for the continuing arms in Design 3, and re-normalising the randomisation probabilities for Designs 4-6.

The updated randomisation probabilities were then used to allocate treatments to the next 50 patients, since RAR or arm dropping was performed every 50 patients in Designs 3-6. Since these allocations might not have matched the allocations that actually occurred in the CAST study data, we needed to resample data for the patients in Designs 3-6. We maintained the original enrolment dates for the CAST patients in the re-execution, and at each enrolment time, we used the current randomisation probabilities to allocate the next patient to a treatment arm. We then randomly sampled a CAST patient for entry into the re-execution that had a matching treatment allocation and was randomised into the original CAST study within ±6 weeks of the re-execution enrolment date. Some of the CAST patients could be resampled several times within a single virtual re-execution of the trial. Once data had been obtained for the next 50 patients then the next interim analysis was performed. This process was repeated for each interim analysis. At each interim analysis the arm allocation probabilities were updated and arm dropping/suspension criteria were checked. Early stopping for efficacy or futility was assessed at the interim analyses performed every 200 patients. To avoid bias, for each design the trial was virtually re-executed 1000 times by drawing data from the CAST study dataset and performing the interim analyses (1000 datasets each with a maximum sample size of N=584 patients).
