# Supplementary material for: Bayesian adaptive designs for multi-arm trials: an orthopaedic case study
Source: Trials. 2020 Jan 14;21:83. doi: 10.1186/s13063-019-4021-0 (PMC6961269; doi:10.1186/s13063-019-4021-0)
Supplement: Supplementary file 2 — Additional file 2. Additional operating characteristics. [file 13063_2019_4021_MOESM2_ESM.docx]

Additional File 2 - Additional Operating Characteristics

**Table A2.1 Additional operating characteristics for Bayesian designs**

| Scenario | Average duration (weeks) | Average sample size (sd) | Proportion stopping early for efficacy | Overall proportion successful | Proportion stopping early for futility | Proportion unsuccessful |
| --- | --- | --- | --- | --- | --- | --- |
| Null (50, 50, 50, 50) |  |  |  |  |  |  |
| Design 1 | 147 | 643 (0) | NA | 0.0022 | NA | 0.9978 |
| Design 2 | 146 | 638 (42) | 0.0063 | 0.0071 | 0.013 | 0.992 |
| Design 3 | 146 | 637 (45) | 0.0025 | 0.0028 | 0.0248 | 0.9954 |
| Design 4 | 146 | 639 (38) | 0.0022 | 0.0023 | 0.0125 | 0.9965 |
| Design 5 | 146 | 640 (34) | 0.0015 | 0.0016 | 0.0134 | 0.9971 |
| Design 6 | 147 | 642 (20) | 0.0117 | 0.023 | 0 | 0.9723 |
|  |  |  |  |  |  |  |
| One arm works, 10 more (50, 50, 50, 60) |  |  |  |  |  |  |
| Design 1 | 147 | 643 (0) | NA | 0.7731 | NA | 0.2269 |
| Design 2 | 112 | 470 (167) | 0.732 | 0.8043 | 0 | 0.1832 |
| Design 3 | 114 | 480 (165) | 0.6919 | 0.8358 | 0.0022 | 0.1489 |
| Design 4 | 111 | 461 (163) | 0.796 | 0.8601 | 0 | 0.1268 |
| Design 5 | 111 | 461 (162) | 0.7909 | 0.8585 | 0 | 0.1269 |
| Design 6 | 94 | 379 (115) | 0.9972 | 0.9986 | 0 | 0.0002 |
|  |  |  |  |  |  |  |
| One arm works, 5 more (50, 50, 50, 55) |  |  |  |  |  |  |
| Design 1 | 147 | 643 (0) | NA | 0.1049 | NA | 0.8951 |
| Design 2 | 142 | 617 (90) | 0.1091 | 0.1454 | 0.0015 | 0.8432 |
| Design 3 | 143 | 624 (80) | 0.0624 | 0.089 | 0.0052 | 0.9007 |
| Design 4 | 143 | 624 (79) | 0.0733 | 0.1008 | 0.0008 | 0.8894 |
| Design 5 | 143 | 625 (78) | 0.0677 | 0.0902 | 0.001 | 0.8995 |
| Design 6 | 136 | 589 (99) | 0.5654 | 0.6853 | 0 | 0.2987 |
|  |  |  |  |  |  |  |
| Better best (50, 55, 60, 65) |  |  |  |  |  |  |
| Design 1 | 147 | 643 (0) | NA | 0.9975 | NA | 0.0025 |
| Design 2 | 110 | 459 (162) | 0.7953 | 0.9977 | 0 | 0.002 |
| Design 3 | 112 | 467 (167) | 0.6843 | 0.9887 | 0.0001 | 0.0094 |
| Design 4 | 108 | 450 (161) | 0.8177 | 0.9984 | 0 | 0.001 |
| Design 5 | 109 | 451 (162) | 0.8069 | 0.9989 | 0 | 0.0008 |
| Design 6 | 113 | 471 (140) | 0.8982 | 0.9386 | 0 | 0.0512 |
|  |  |  |  |  |  |  |
| One worse, others work (50, 45, 55, 60) |  |  |  |  |  |  |
| Design 1 | 147 | 643 (0) | NA | 0.7772 | NA | 0.2228 |
| Design 2 | 120 | 511 (160) | 0.6341 | 0.8056 | 0 | 0.1865 |
| Design 3 | 120 | 509 (160) | 0.6123 | 0.815 | 0.0005 | 0.1756 |
| Design 4 | 119 | 503 (159) | 0.6872 | 0.8421 | 0 | 0.1469 |
| Design 5 | 119 | 501 (159) | 0.6856 | 0.8412 | 0 | 0.148 |
| Design 6 | 113 | 473 (140) | 0.8972 | 0.9384 | 0 | 0.0535 |
|  |  |  |  |  |  |  |
| All work, two similar (50, 55, 60, 60) |  |  |  |  |  |  |
| Design 1 | 147 | 643 (0) | NA | 0.8979 | NA | 0.1021 |
| Design 2 | 136 | 588 (122) | 0.2701 | 0.908 | 0 | 0.0899 |
| Design 3 | 135 | 584 (126) | 0.2692 | 0.8915 | 0.0004 | 0.1041 |
| Design 4 | 136 | 588 (121) | 0.2744 | 0.9158 | 0 | 0.0821 |
| Design 5 | 136 | 589 (120) | 0.2744 | 0.9179 | 0 | 0.0792 |
| Design 6 | 137 | 592 (97) | 0.5493 | 0.6788 | 0 | 0.3033 |

**Table A2.2. Arm dropping proportions and average time each arm was dropped for Design 3**

| Scenario^a^ | Boot | | Brace | | Below-knee cast | | |
| --- | --- | --- | --- | --- | --- | --- | --- |
|  | **Proportion of simulations arm dropped** | **Average time to arm drop (sd)^b^** | **Proportion of simulations arm dropped** | **Average time to arm drop (sd) ^b^** | **Proportion of simulations arm dropped** | **Average time to arm drop (sd) ^b^** | **Average duration of trial (sd) ^b^** |
| Null (50, 50, 50, 50) | 0.5103 | 48.50 (26.81) | 0.5274 | 48.99 (27.64) | 0.5246 | 48.53 (27.52) | 145.88 (10.24) |
| One arm works, 10 more (50, 50, 50, 60) | 0.9839 | 39.52 (15.80) | 0.9841 | 39.34 (15.59) | 0.0279 | 30.37 (4.77) | 114.22 (33.23) |
| One arm works, 5 more (50, 50, 50, 55) | 0.8687 | 50.35 (27.26) | 0.8762 | 50.38 (27.42) | 0.1303 | 35.31 (12.85) | 143.15 (16.84) |
| Better best (50, 55, 60, 65) | 0.9938 | 37.30 (14.62) | 0.8631 | 54.41 (28.90) | 0.0763 | 34.30 (11.51) | 111.72 (33.69) |
| One worse, others work (50, 45, 55, 60) | 0.9998 | 31.30 (6.42) | 0.873 | 56.71 (29.33) | 0.0603 | 35.75 (13.22) | 120.18 (32.37) |
| All work, two same (50, 55, 60, 60) | 0.9582 | 45.41 (23.94) | 0.3636 | 49.88 (28.39) | 0.3663 | 49.74 (28.47) | 135.13 (25.70) |

^a^Each row represents a different scenario where the assumed FAOS QoL score is given in brackets as tubular bandage, boot, brace and below-knee cast score; ^b^Time in weeks

**Table A2.3 Proportion of simulations where the true best arm was correctly declared to be the best arm**

|  | Design 1 | Design 2 | Design 3 | Design 4 | Design 5 | Design 6 |
| --- | --- | --- | --- | --- | --- | --- |
| One arm works, 10 more (50, 50, 50, 60) | 1 | 0.9998 | 0.98 | 0.999 | 0.9999 | 1 |
| One arm works, 5 more (50, 50, 50, 55) | 0.9562 | 0.9565 | 0.8712 | 0.9591 | 0.9475 | 0.9802 |
| Better best (50, 55, 60, 65) | 0.976 | 0.9711 | 0.9565 | 0.9792 | 0.9745 | 0.9802 |
| One worse, others work (50, 45, 55, 60) | 0.9759 | 0.9721 | 0.9379 | 0.9806 | 0.9788 | 0.998 |
| All work, two similar (50, 55, 60, 60) | 0.9955 | 0.9954 | 0.9812 | 0.9946 | 0.9938 | 0.9987 |
