# Supplementary material for: Bayesian adaptive designs for multi-arm trials: an orthopaedic case study
Source: Trials. 2020 Jan 14;21:83. doi: 10.1186/s13063-019-4021-0 (PMC6961269; doi:10.1186/s13063-019-4021-0)
Supplement: Supplementary file 3 — Additional file 3. Faster recruitment rates. [file 13063_2019_4021_MOESM3_ESM.docx]

Additional File 3 - Faster recruitment rates

**Table A3.1. Effect of varying recruitment rate on average sample size and probability of trial success for Bayesian designs**

|  | 5 patients/week | | 25 patients/week | | 56 patients/week | |
| --- | --- | --- | --- | --- | --- | --- |
| Scenario | Average sample size (sd) | Overall proportion successful | Average sample size (sd) | Overall proportion successful | Average sample size (sd) | Overall proportion successful |
| Null (50, 50, 50, 50) |  |  |  |  |  |  |
| Design 1 | 643 (0) | 0.0022 | 643 (0) | 0.0015 | 643 (0) | 0.0016 |
| Design 2 | 638 (42) | 0.0071 | 642 (15) | 0.0017 | 643 (0) | 0.0022 |
| Design 3 | 637 (45) | 0.0028 | 642 (15) | 0.0013 | 643 (0) | 0.0024 |
| Design 4 | 639 (38) | 0.0023 | 642 (16) | 0.0015 | 643 (0) | 0.0018 |
| Design 5 | 640 (34) | 0.0016 | 642 (16) | 0.0003 | 643 (0) | 0.0014 |
| Design 6 | 642 (20) | 0.023 | 643 (9) | 0.0238 | 643 (0) | 0.032 |
|  |  |  |  |  |  |  |
| One arm works, 10 more (50, 50, 50, 60) |  |  |  |  |  |  |
| Design 1 | 643 (0) | 0.7731 | 643 (0) | 0.7838 | 643 (0) | 0.7817 |
| Design 2 | 470 (167) | 0.8043 | 596 (68) | 0.7813 | 643 (0) | 0.7846 |
| Design 3 | 480 (165) | 0.8358 | 602 (66) | 0.7939 | 643 (0) | 0.7702 |
| Design 4 | 461 (163) | 0.8601 | 595 (69) | 0.8093 | 643 (0) | 0.7828 |
| Design 5 | 461 (162) | 0.8585 | 595 (69) | 0.809 | 643 (0) | 0.7875 |
| Design 6 | 379 (115) | 0.9986 | 585 (71) | 0.9991 | 643 (0) | 0.9979 |
|  |  |  |  |  |  |  |
| One arm works, 5 more (50, 50, 50, 55) |  |  |  |  |  |  |
| Design 1 | 643 (0) | 0.1049 | 643 (0) | 0.1159 | 643 (0) | 0.1067 |
| Design 2 | 617 (90) | 0.1454 | 636 (33) | 0.1202 | 643 (0) | 0.1103 |
| Design 3 | 624 (80) | 0.089 | 637 (29) | 0.0812 | 643 (0) | 0.1014 |
| Design 4 | 624 (79) | 0.1008 | 635 (33) | 0.097 | 643 (0) | 0.109 |
| Design 5 | 625 (78) | 0.0902 | 636 (33) | 0.0848 | 643 (0) | 0.1076 |
| Design 6 | 589 (99) | 0.6853 | 637 (31) | 0.6295 | 643 (0) | 0.6026 |
|  |  |  |  |  |  |  |
| Better best (50, 55, 60, 65) |  |  |  |  |  |  |
| Design 1 | 643 (0) | 0.9975 | 643 (0) | 0.9976 | 643 (0) | 0.9984 |
| Design 2 | 459 (162) | 0.9977 | 593 (69) | 0.9974 | 643 (0) | 0.9974 |
| Design 3 | 467 (167) | 0.9887 | 597 (68) | 0.9897 | 643 (0) | 0.9837 |
| Design 4 | 450 (161) | 0.9984 | 592 (69) | 0.9988 | 643 (0) | 0.9982 |
| Design 5 | 451 (162) | 0.9989 | 592 (69) | 0.9986 | 643 (0) | 0.9984 |
| Design 6 | 471 (140) | 0.9386 | 615 (58) | 0.9032 | 643 (0) | 0.8432 |
|  |  |  |  |  |  |  |
| One worse, others work (50, 45, 55, 60) |  |  |  |  |  |  |
| Design 1 | 643 (0) | 0.7772 | 643 (0) | 0.7872 | 643 (0) | 0.7867 |
| Design 2 | 511 (160) | 0.8056 | 609 (62) | 0.7877 | 643 (0) | 0.7877 |
| Design 3 | 509 (160) | 0.815 | 611 (61) | 0.774 | 643 (0) | 0.7608 |
| Design 4 | 503 (159) | 0.8421 | 608 (63) | 0.8032 | 643 (0) | 0.7867 |
| Design 5 | 501 (159) | 0.8412 | 608 (63) | 0.8057 | 643 (0) | 0.7897 |
| Design 6 | 473 (140) | 0.9384 | 615 (58) | 0.8997 | 643 (0) | 0.8431 |
|  |  |  |  |  |  |  |
| All work, two similar (50, 55, 60, 60) |  |  |  |  |  |  |
| Design 1 | 643 (0) | 0.8979 | 643 (0) | 0.9042 | 643 (0) | 0.9037 |
| Design 2 | 588 (122) | 0.908 | 629 (44) | 0.9016 | 643 (0) | 0.8998 |
| Design 3 | 584 (126) | 0.8915 | 628 (45) | 0.8746 | 643 (0) | 0.8658 |
| Design 4 | 588 (121) | 0.9158 | 629 (43) | 0.9076 | 643 (0) | 0.9048 |
| Design 5 | 589 (120) | 0.9179 | 629 (44) | 0.9087 | 643 (0) | 0.9048 |
| Design 6 | 592 (97) | 0.6788 | 637 (31) | 0.6354 | 643 (0) | 0.604 |

Faster recruitment rates tended to increase the average sample size and decrease the probability of having a successful trial/power since the recruitment was often “too fast” to enable the decisions from the interim analyses to be implemented (i.e., all of the patients might have been recruited before some of the interim analyses could occur).
