# Supplementary material for: Bayesian adaptive designs for multi-arm trials: an orthopaedic case study
Source: Trials. 2020 Jan 14;21:83. doi: 10.1186/s13063-019-4021-0 (PMC6961269; doi:10.1186/s13063-019-4021-0)
Supplement: Supplementary file 4 — Additional file 4. Virtual execution of Bayesian designs. [file 13063_2019_4021_MOESM4_ESM.docx]

Additional File 4 – Virtual execution of Bayesian designs

# Design 1

When a single re-execution was performed on the actual CAST data (i.e., no resampling of CAST data) using Bayesian Design 1, the trial was found to be successful. The posterior probability of having a MCID of 8 between the best arm and control was 0.87. The posterior probability of the best arm being superior to the control was approximately 1.00. The posterior probabilities of being the best arm were 0, 0.08, 0.40, and 0.53 for tubular bandage, Bledsoe boot, ankle brace, and below-knee cast, respectively. The Bayesian analysis of the CAST data gave a posterior mean estimate of 53.42 (95% Highest Density Interval (HDI): 49.11, 57.89) for the FAOS QoL for the tubular bandage. The posterior mean difference between the Bledsoe boot and tubular bandage was 6.90 (95% HDI: 0.75, 12.91). The posterior mean difference between the ankle brace and tubular bandage was 9.47 (95% HDI 3.38, 15.64). The posterior mean difference between the below-knee cast and tubular bandage was 10.02 (95% HDI: 3.76, 16.22).

# Design 2

**Table A4.1. Summary of posterior probabilities at each interim analysis for Design 2 when performing virtual executions of the CAST study using the actual CAST data (no resampling)**

| Analysis | Posterior probability of having a MCID | Posterior probability best arm is the best | Posterior probability best arm is superior to tubular bandage |
| --- | --- | --- | --- |
| Interim analysis 1 (200 patients) | 0.94 | 0.75 | 1 |
| Interim analysis 2 (400 patients) | 0.95 | 0.50 | 1 |

Although the probability of having a MCID exceeded the stopping boundary at each interim analysis, there wasn’t a sufficiently high probability that the best dose was indeed the best dose and so the trial did not stop early for efficacy.

**Table A4.2 Summary of posterior probabilities at each interim analysis for Design 2 over the 1000 virtual re-executions of the CAST study**

| Analysis | Median (IQR) posterior probability of having a MCID | Median (IQR) posterior probability best arm is the best | Median (IQR) posterior probability best arm is superior to tubular bandage |
| --- | --- | --- | --- |
| Interim analysis 1 (200 patients) | 0.95 (0.85, 0.99) | 0.73 (0.57, 0.89) | 1 (1,1) |
| Interim analysis 2 (400 patients) | 0.94 (0.83, 0.98) | 0.61 (0.51, 0.74) | 1 (1,1) |

Although the probability of having a MCID exceeded the stopping boundary at each interim analysis, there wasn’t a sufficiently high probability that the best dose was indeed the best dose in most of the re-executions and so the trial did not often stop early for success.

# Design 3

The frequency of arm dropping in the virtual re-executions is summarised in Table A4.3.

**Table A4.3. Cumulative proportion of arm dropping across the 1000 virtual re-executions of the CAST study using Bayesian Design 3**

| Interim analysis | Boot | Brace | Below-knee Cast |
| --- | --- | --- | --- |
| 1^a^ | 0 | 0 | 0 |
| 2 | 0.12 | 0.08 | 0.14 |
| 3 | 0.43 | 0.39 | 0.14 |
| 4 | 0.49 | 0.37 | 0.16 |
| 5 | 0.57 | 0.40 | 0.16 |
| 6 | 0.58 | 0.44 | 0.19 |
| 7 | 0.59 | 0.46 | 0.32 |
| 8 | 0.59 | 0.48 | 0.32 |
| 9 | 0.61 | 0.50 | 0.34 |
| 10 | 0.66 | 0.50 | 0.36 |
| 11 | 0.70 | 0.50 | 0.38 |

^a^note that interim analysis 1 is based on 1 run since the actual CAST data was used for this analysis

**
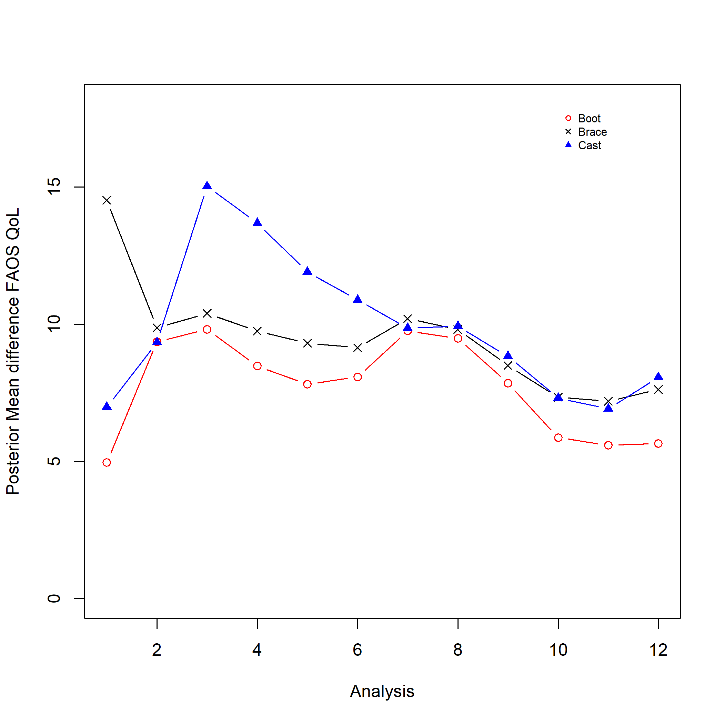
**

**Figure A4.1. Median over the 1000 virtual re-executions of CAST of the estimates of the posterior mean difference in FAOS QoL score between each arm and tubular bandage (control) at each interim analysis using Bayesian Design 3**

**
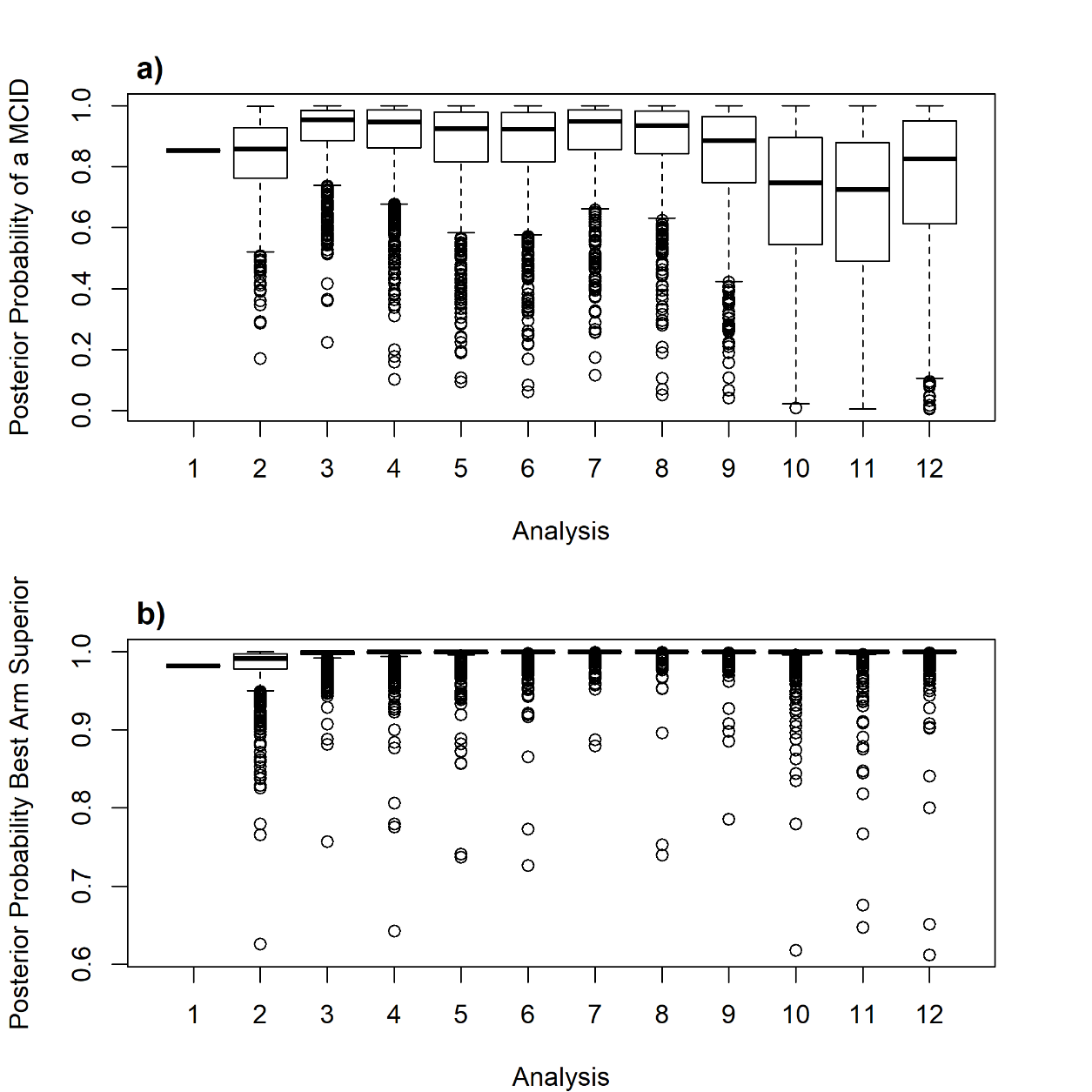
**

**Figure A4.2. Re-execution of the CAST study using Bayesian Design 3 showing boxplots of posterior probabilities at each analysis over the 1000 replications of the trial a)** Boxplot of the posterior probability of having a MCID at each analysis over the re-executions; **b)** Boxplot of the posterior probability that the best arm is superior to the tubular bandage at each analysis over the re-executions. The 12^th^ analysis is the final analysis. Note that interim analysis 1 is based on 1 run since the actual CAST data was used for this analysis and so consists of a single posterior probability.

# Design 4


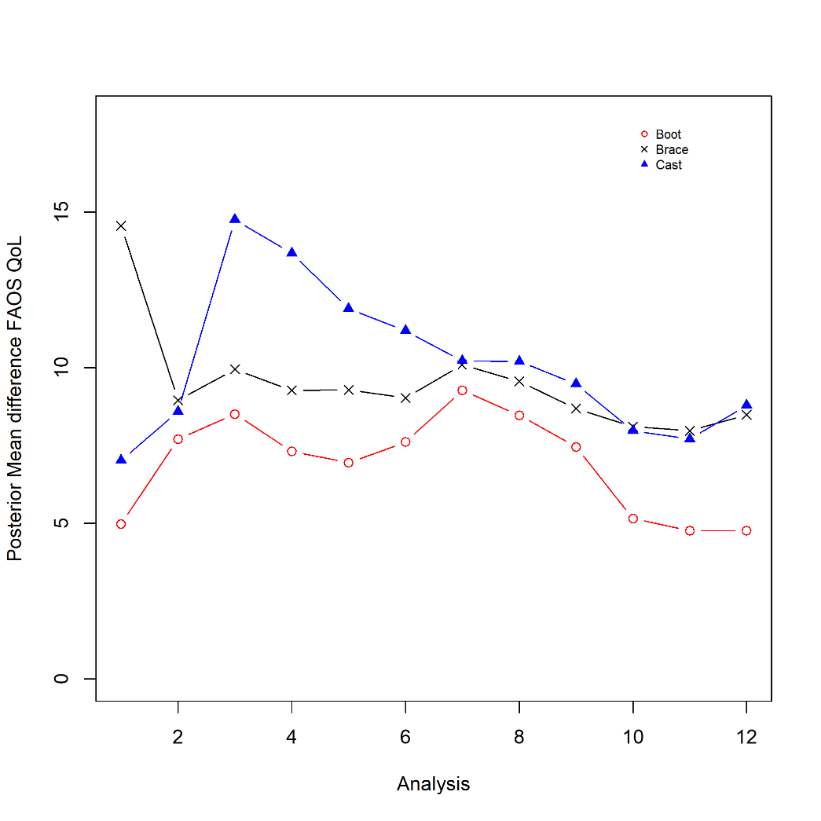


**Figure A4.3. Median over the 1000 virtual re-executions of CAST of the estimates of the posterior mean difference in FAOS QoL score between each arm and tubular bandage (control) at each interim analysis using Bayesian Design 4**

**
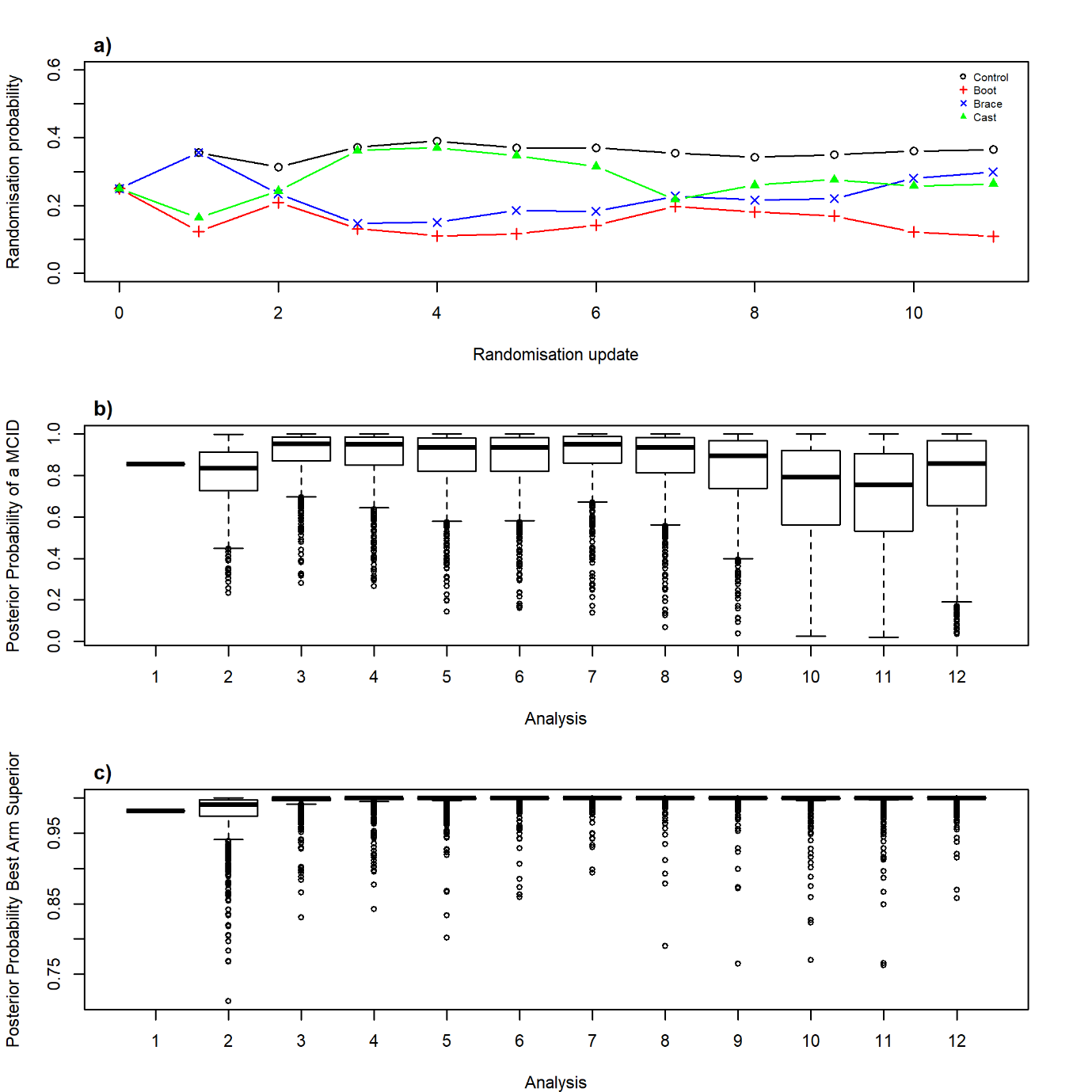
**

**Figure A4.4. Re-execution of the CAST study using Bayesian Design 4 showing the randomisation and posterior probabilities at each analysis over the 1000 replications of the trial** a) Median randomisation probabilities for each arm at each allocation update; b) Boxplot of the posterior probability of having a MCID at each analysis over the re-executions; c) Boxplot of the posterior probability that the best arm is superior to the tubular bandage at each analysis over the re-executions. The 12^th^ analysis is the final analysis. Note that interim analysis 1 is based on 1 run since the actual CAST data was used for this analysis and so consists of a single posterior probability.

**
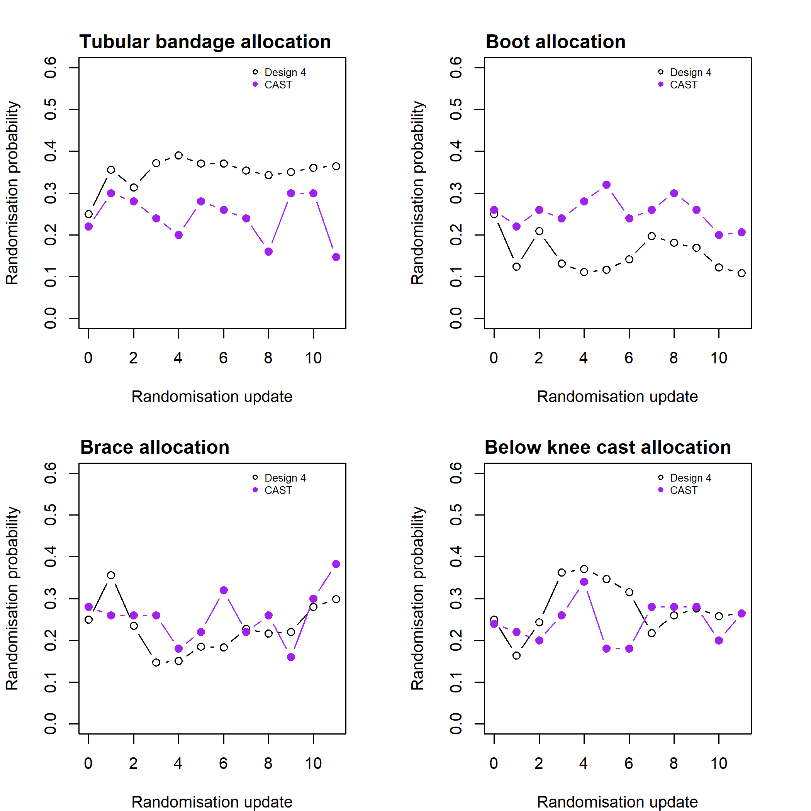


Figure A4.5. Actual proportions allocated in CAST study (closed purple circles) compared to median RAR allocation probabilities for the next 50 patients at each allocation update over the 1000 simulated trials for each arm in Bayesian Design 4 (open circles).** Randomisation in the original CAST study was stratified by centre.

From Figure A4.5 it can be seen that Bayesian Design 4 allocated a higher proportion to tubular bandage at each interim analysis as this was always matched to the best arm allocation.

# Design 5


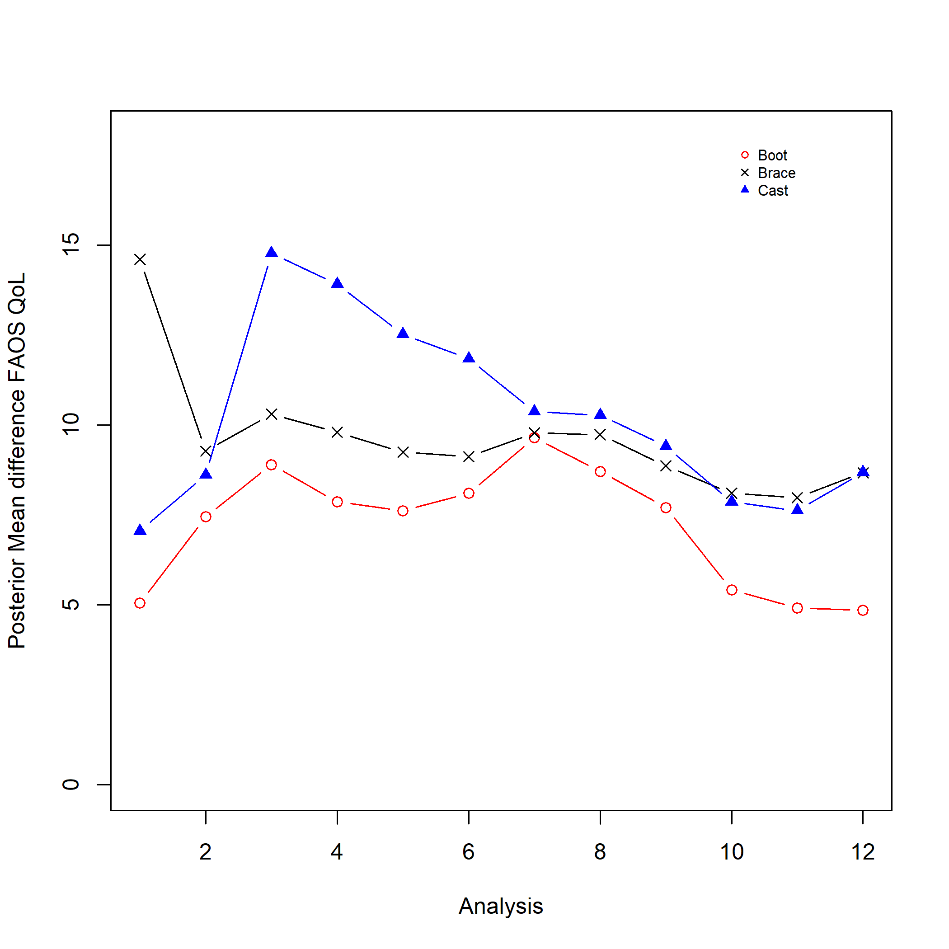


**Figure A4.6. Median over the 1000 virtual re-executions of CAST of the estimates of the posterior mean difference in FAOS QoL score between each arm and tubular bandage (control) at each interim analysis using Bayesian Design 5**


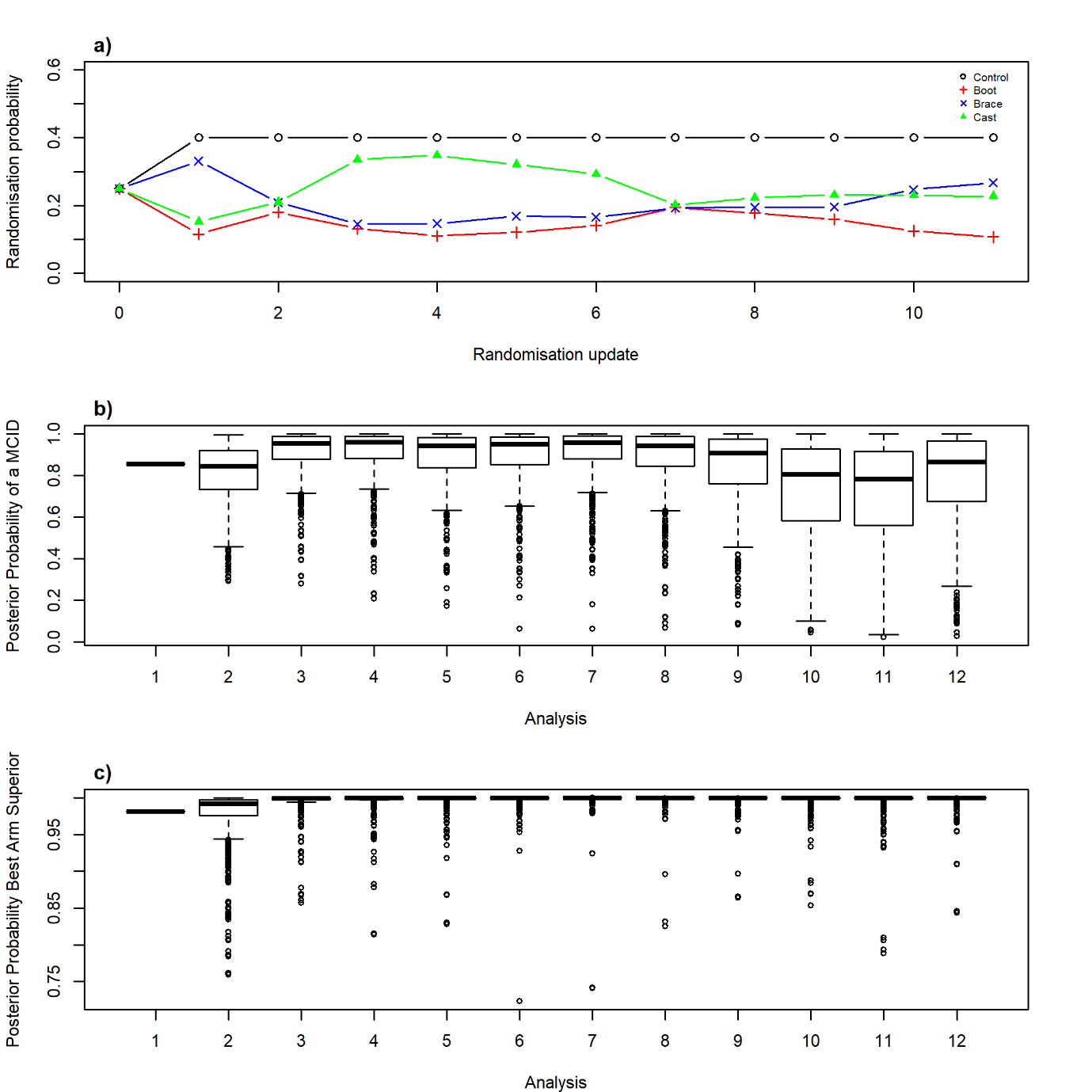


**Figure A4.7. Re-execution of the CAST study using Bayesian Design 5 showing the randomisation and posterior probabilities at each analysis over the 1000 replications of the trial** a) Median randomisation probabilities for each arm at each allocation update; b) Boxplot of the posterior probability of having a MCID at each analysis over the re-executions; c) Boxplot of the posterior probability that the best arm is superior to the tubular bandage at each analysis over the re-executions. The 12^th^ analysis is the final analysis. Note that interim analysis 1 is based on 1 run since the actual CAST data was used for this analysis and so consists of a single posterior probability.


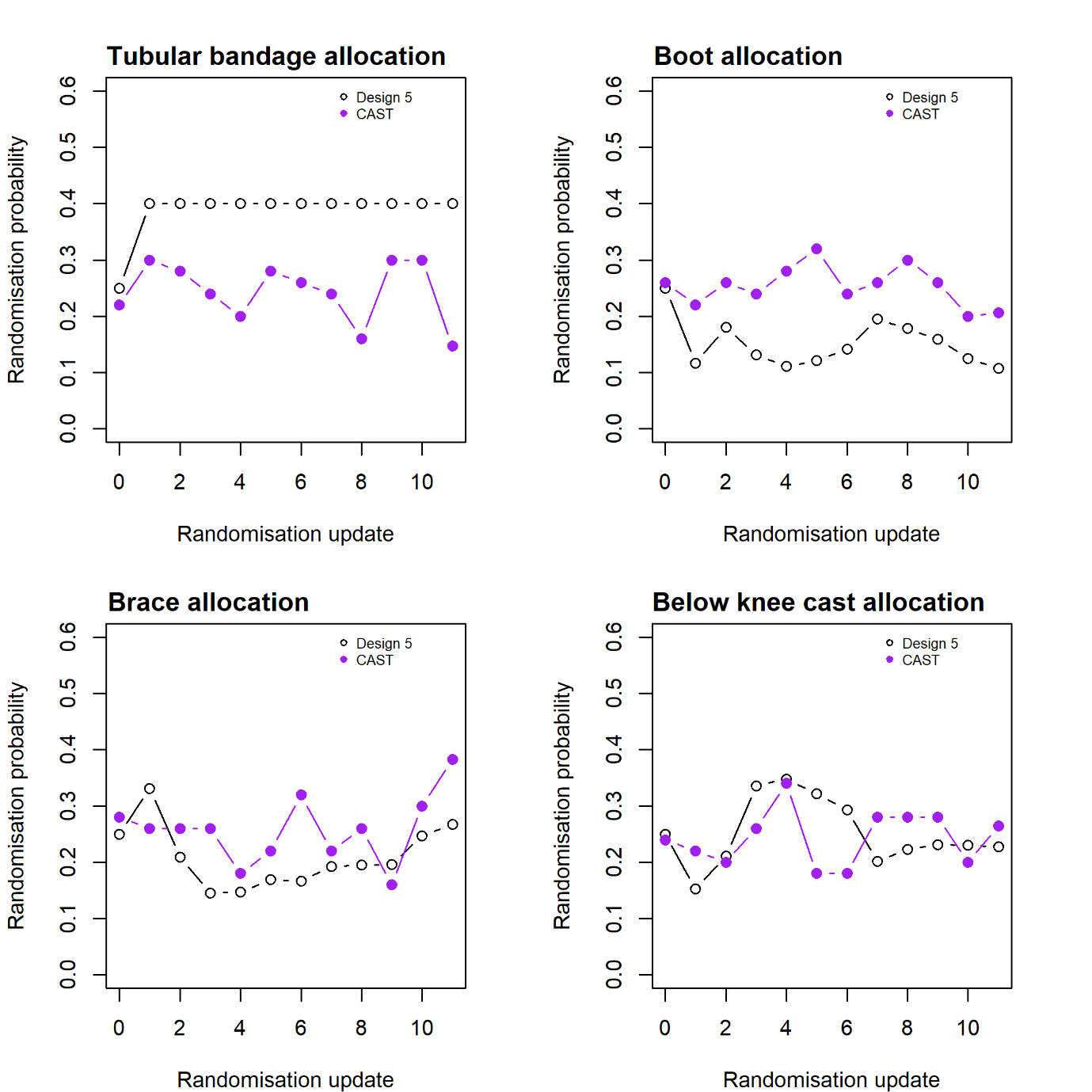


**Figure A4.8. Actual proportions allocated in CAST study (closed purple circles) compared to median RAR allocation probabilities for the next 50 patients at each allocation update over the 1000 simulated trials for each arm in Bayesian Design 5 (open circles).** Randomisation in the original CAST study was stratified by centre.

## **Design 6**
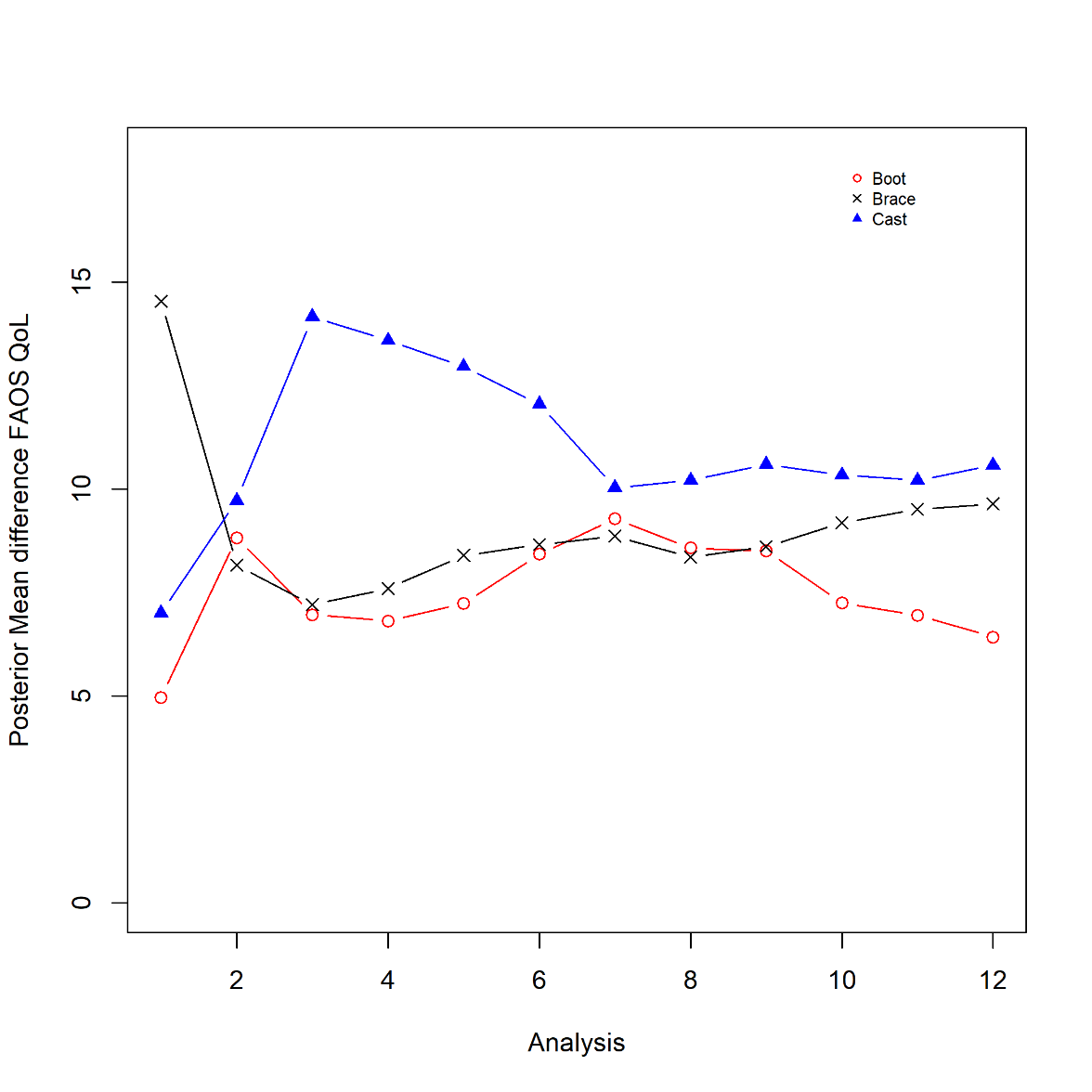


**Figure A4.9. Median over the 1000 virtual re-executions of CAST of the estimates of the posterior mean difference in FAOS QoL score between each arm and tubular bandage (control) at each interim analysis using Bayesian Design 6**


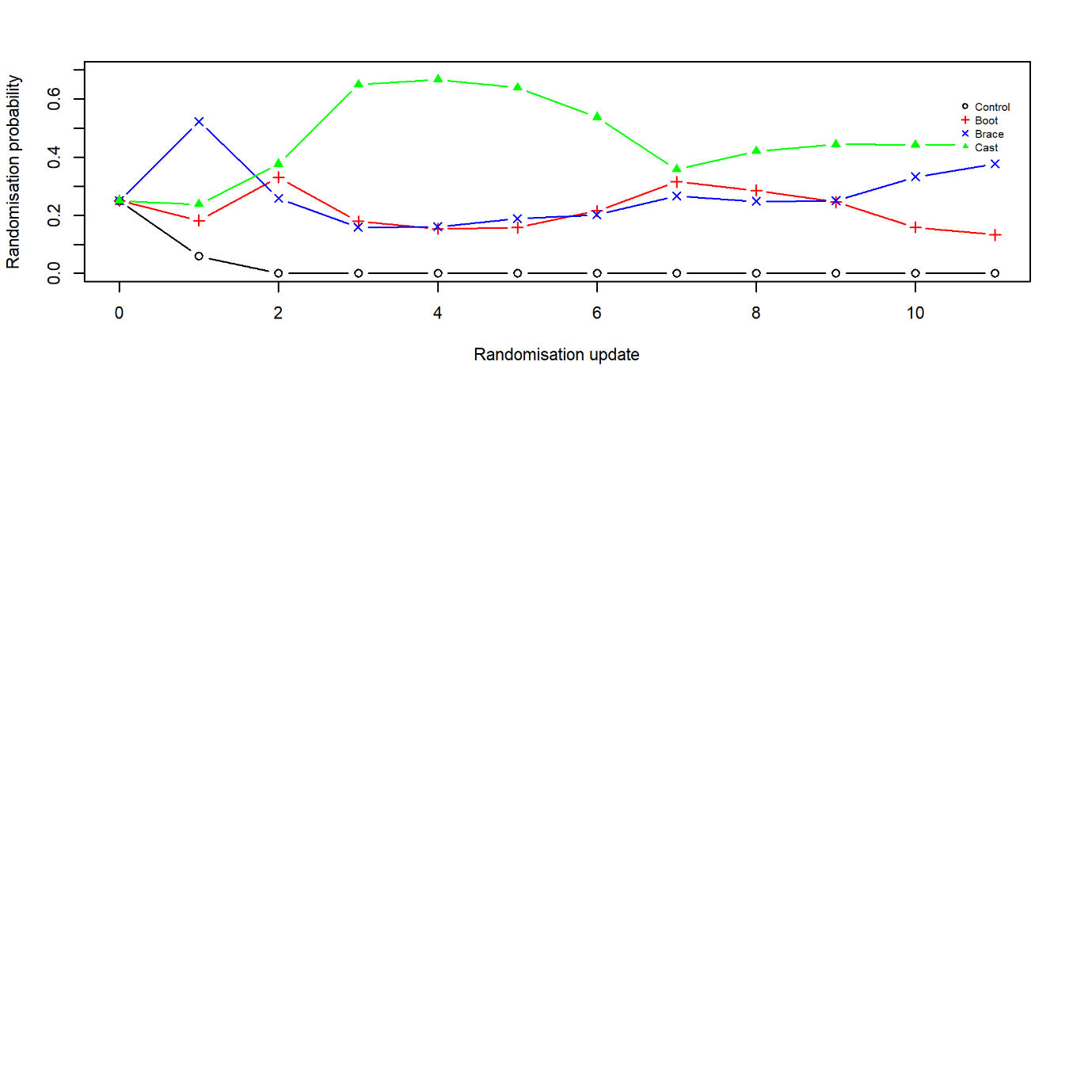


**Figure A4.10. Re-execution of the CAST study using Bayesian design 6 showing the median randomisation probabilities for each arm at each allocation update over the 1000 replications of the trial.** The randomisation probabilities are proportional to the probability of the arm being the best arm.


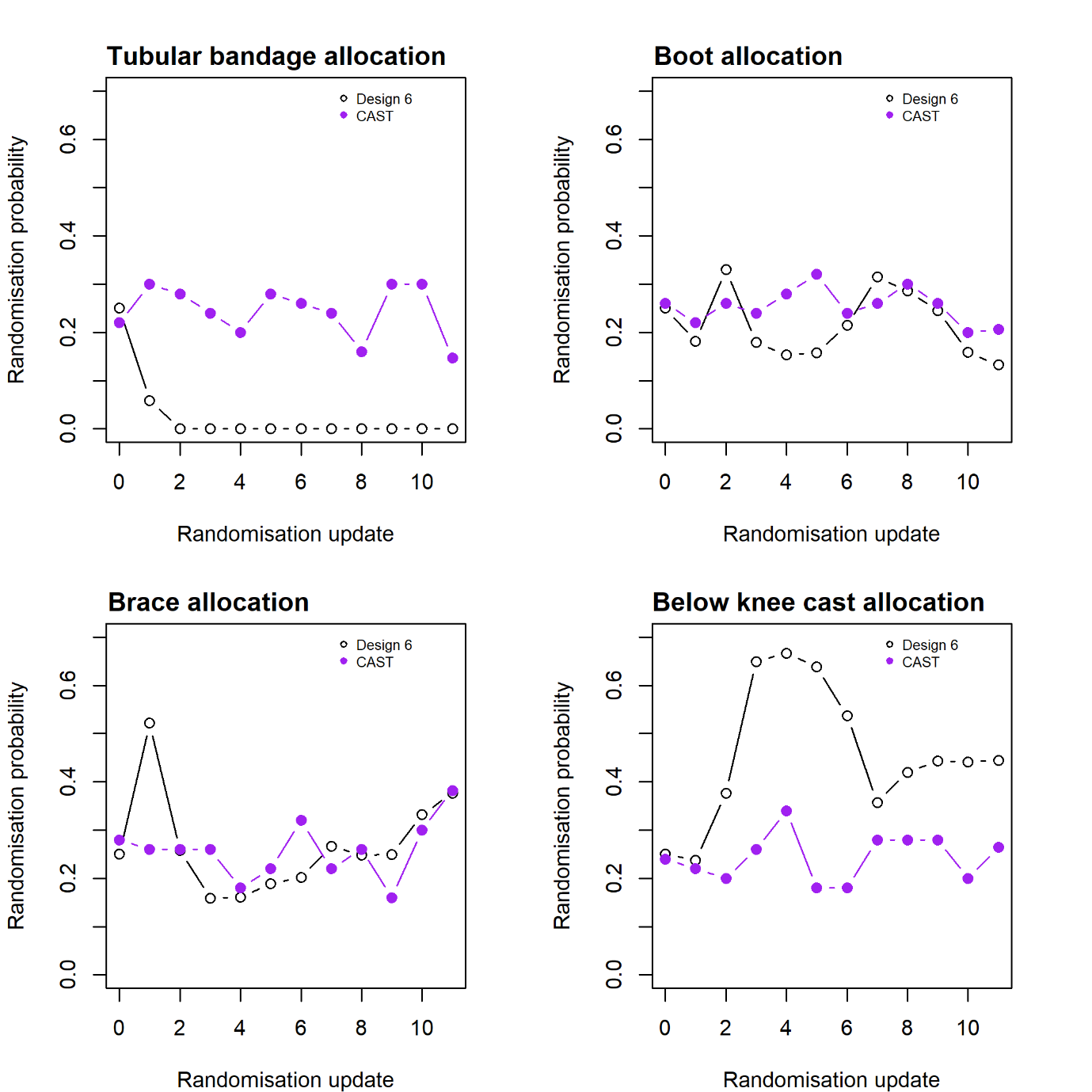

 **Figure A4.11. Actual proportions allocated in CAST study (closed purple circles) compared to median RAR allocation probabilities for the next 50 patients at each allocation update over the 1000 simulated trials for each arm in Bayesian Design 6 (open circles).** Randomisation in the original CAST study was stratified by centre.

From Figure A4.11 it can be seen that the allocation to the control was quite low at each interim analysis, and that Bayesian design 6 often allocated a higher proportion of patients to the intervention arms than the CAST study, particularly for the below-knee cast.

From Figures A4.4a, A4.7a, and A4.10 it can be seen that Bayesian adaptive designs 4-6 often had different allocation probability patterns, with some arms being given much higher allocations at certain interim analyses. From Figures A4.5, A4.8, and A4.11 it can be seen that the Bayesian adaptive designs with RAR often had quite different allocations to the CAST study, depending on which arm was “the best” at that interim analysis.
